# Anodic coupling of carboxylic acids to electron-rich double bonds: A surprising non-Kolbe pathway to lactones

**DOI:** 10.3762/bjoc.9.186

**Published:** 2013-08-09

**Authors:** Robert J Perkins, Hai-Chao Xu, John M Campbell, Kevin D Moeller

**Affiliations:** 1 Washington University in Saint Louis, Saint Louis, Missouri 63130, United States

**Keywords:** carboxylic acid, cyclization, electrolysis, free radical, kolbe, radical cation

## Abstract

Carboxylic acids have been electro-oxidatively coupled to electron-rich olefins to form lactones. Kolbe decarboxylation does not appear to be a significant competing pathway. Experimental results indicate that oxidation occurs at the olefin and that the reaction proceeds through a radical cation intermediate.

## Introduction

**Scheme 1:**
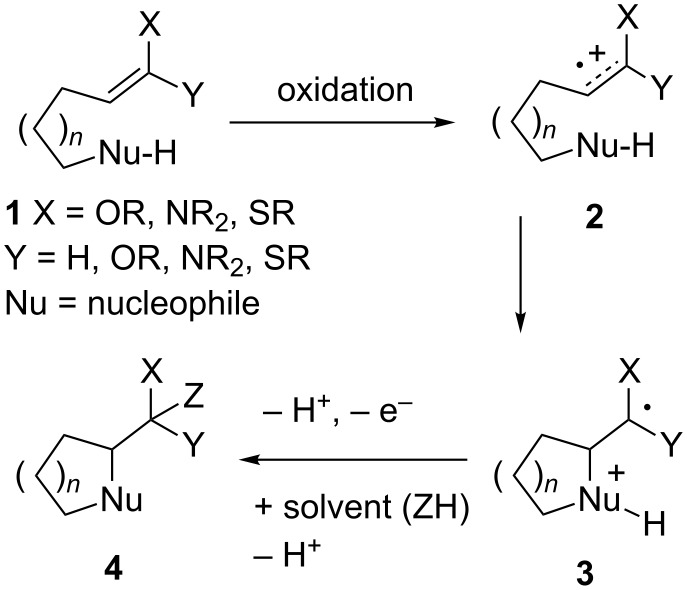
General scheme for anodic cyclization reactions.

Anodic cyclization reactions of the general type shown in Scheme 1 can provide a powerful method for the synthesis of a variety of ring systems [1–2]. The reactions effectively reverse the polarity of an electron-rich functional group and in so doing open up entirely new opportunities for bond formation. In addition, the oxidative reactions lead to products that either preserve or increase the level of functionality found in the initial substrate. This provides synthetic handles for further development of the cyclic products generated.

Such reactions can be used to build a variety of fused, bridged, and spirocyclic carbocyclic systems [3], cyclic amino acid derivatives [4], cyclic ethers [5–6], and lactones [7–8]. In most of these examples, the reactions can be viewed as arising from an oxidation that forms an olefinic radical cation that is then rapidly trapped by a nucleophile. This triggers a cascade of reactions that typically involve the loss of two protons (or a silyl group), a second oxidation of a radical intermediate, and solvent trapping. This reaction cascade leads to formation of the final product.

**Scheme 2:**
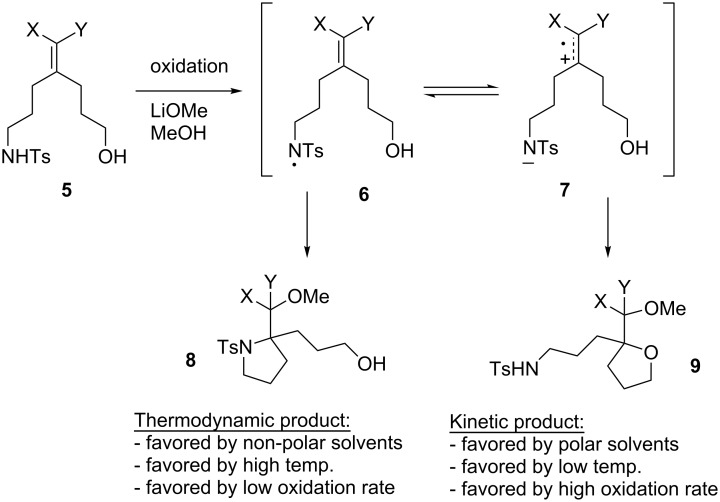
Anodic cyclization competition study.

A combination of computational studies and competition experiments has begun to identify both cyclization reactions that proceed through different mechanistic pathways and cyclization reactions where the cascade of reactions downstream from the cyclization plays an important role in product formation [9]. For example, consider the competition experiment highlighted in Scheme 2. In this experiment, an electron-rich olefin was coupled to one of two competing nucleophiles, a sulfonamide and an alcohol (**5**). When the oxidation was run with 2,6-lutidine as a base (not shown), the reaction led to the formation of a radical cation from the electron-rich olefin followed by trapping by the alcohol nucleophile to form product **9**. No product from sulfonamide trapping (**8**) was observed. However, when the reaction was run under more basic conditions (0.5 equivalents of lithium methoxide) cyclic voltammetry data indicated that the oxidative cyclizations were initiated by the oxidation of sulfonamide anion. This led to initial formation of N-localized radical **6**. Depending on the reaction conditions, the experiment favoured either a sulfonamide or alcohol based cyclization. Density functional theory (DFT) calculations suggested that sulfonamide based cyclizations proceed by addition of the N-localized radical to the electron-rich double bond, a reaction that led to product **8**. In a competitive process, an intramolecular electron-transfer reaction occurred that led to formation of a radical cation from the olefin **7** and regeneration of the sulfonamide anion. Formation of a radical cation from the olefin led to the possibility of alcohol trapping and the formation of cyclic ether product **9**. Experimental data suggested that the alcohol trapping pathway provided the kinetic product but that the cyclization was reversible. The sulfonamide radical pathway afforded the thermodynamic product. In a very interesting development, we found that the reaction could be shifted toward the alcohol-trapping product by increasing the current passed through the cell. The increased current appeared to accelerate the removal of a second electron from an initial cyclic product like **3** (Scheme 1), a change that reduced the reversibility of the cyclization.

**Scheme 3:**
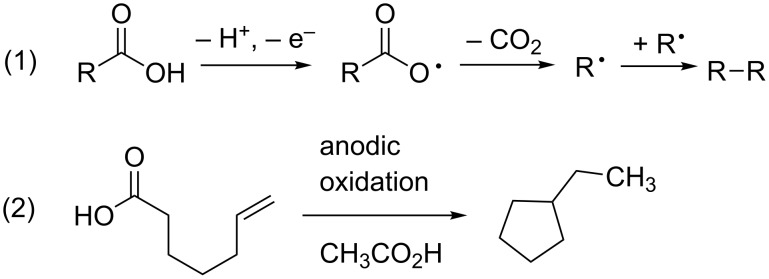
Kolbe electrolysis reactions.

Clearly, the use of basic reaction conditions with the acidic substrate led to dramatic mechanistic changes to our early, simplified view of the reactions. It is tempting to suggest that similar situations would arise with other acidic substrates. For example, would the oxidation of a substrate having a carboxylic acid and an electron-rich olefin lead to a carboxy radical pathway or a radical cation type reaction? To date, we have been hesitant to try such an experiment, and in fact we typically avoid substrates with carboxylic acid functional groups [5]. This action was taken because of the well-known Kolbe electrolysis reaction (Scheme 3) [10–11]. In the Kolbe electrolysis (Scheme 3, reaction 1), a carboxylic acid is oxidized. A decarboxylation reaction then leads to the formation of a radical that is subsequently trapped by a second radical formed in solution. The reaction has been used to form dimers [12], as well as in some cases cyclic products (Scheme 3, reaction 2) [13–15].

However, the chemistry highlighted in Scheme 2 suggests that a Kolbe-type decarboxylation reaction might not interfere at all with an oxidative coupling reaction between a carboxylic acid and an electron-rich olefin. Examples in the literature of the coupling of carboxylic acids to aromatic rings, though limited in scope, were also encouraging [16–20].

**Scheme 4:**
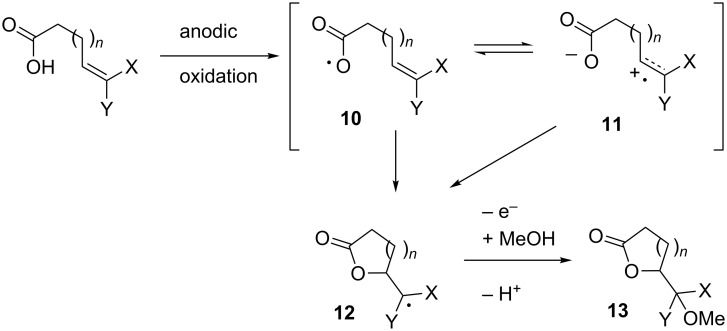
Oxidative coupling between a carboxylic acid and electron-rich olefin.

There are three mechanistic possibilities which would allow for a successful reaction. First, if the carboxy radical **10** is formed, then it may simply add to the electron-rich olefin to give **12** faster than it undergoes a decarboxylation reaction (Scheme 4).

Second, it is possible for a carboxy radical like **10** to undergo an intramolecular electron-transfer reaction analogous to that observed in the case of the sulfonamide-based radical. One might suspect the electron-transfer to form radical cation **11** to be even more favorable in the present case due to the stability of the resulting carboxylate anion. The electron-transfer would minimize the risk of decarboxylation and lead to carboxylate trapping of the radical cation to again form the cyclic radical **12**.

A third mechanistic possibility is a direct oxidation of the elctron-rich olefin to form radical cation **11** followed by carboxylate trapping. All three mechanistic variations would then require a successful single-electron oxidation of **12** to ultimately lead to **13**, the product of a direct oxidative coupling between the carboxylic acid moiety and the electron-rich olefin.

We report here that this is the case and that the direct oxidative coupling of a carboxylic acid and an electron-rich olefin can be accomplished in good yield. In all cases, the reactions appear to proceed through an olefinic radical cation intermediate.

## Results and Discussion

### Initial cyclization studies

The compatibility of the carboxylic acid with the anodic cyclization was initially studied by examining the anodic oxidation of substrates **14a–c** (Table 1). The oxidation of **14a** nicely afforded the five-membered ring product using either lithium methoxide or 2,6-lutidine as a base. Clearly, decarboxylation of the carboxylate anion was not a problem. In fact, cyclic voltammetry data suggest that the reaction originated from an oxidation of the ketene dithioacetal. The oxidation potential (*E*_p/2_) for **14a** was measured to be +0.68 V versus Ag/AgCl. For comparison, the oxidation potential measured for 10-undecenoic acid was +1.91 V versus Ag/AgCl in DMF solvent and +1.85 V versus Ag/AgCl in acetonitrile. When 0.5 equivalents of benzyltrimethylammonium hydroxide was added to the cyclic voltammetry solutions in order to generate the carboxylate and mimic the preparative oxidation conditions used for the reaction originating from **14a**, the oxidation potential of the 10-undecenoic acid fell to 1.36–1.40 V versus Ag/AgCl for the DMF solution and 1.38 V versus Ag/AgCl for the acetonitrile solution. The carboxylate was only partially soluble in acetonitrile. While the formation of the carboxylate significantly lowered the oxidation potential of the acid moiety, it did not lower it close to the oxidation potential measured for substrate **14a** or even the oxidation potential of a ketene dithioacetal (ca. +1.06 V versus Ag/AgCl) [21]. Hence, the oxidation potential measured for **14a** is most consistent with a fast cyclization originating from oxidation of the olefin. Fast cyclization reactions are known to significantly lower the oxidation potential of a ketene dithioacetal. For example, the trapping of a ketene dithioacetal derived radical cation by an amine was shown to lower the oxidation potential measured for the ketene dithioacetal by 460 mV [21], a value consistent with the potential measured here for **14a**.

**Table 1:** Anodic coupling of ketene dithioacetals and carboxylic acids.

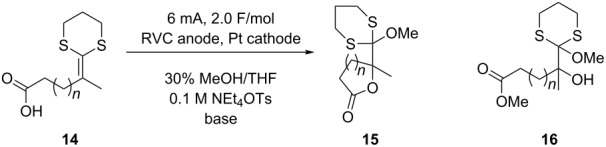				

Substrate	*n*	*E*_p/2_^a^	Base (0.5 equiv)	Yield (**15**)

**14a**	1	0.68	LiOMe	87%
**14a**	1		2,6-lutidine	74%
**14b**	2	0.71	LiOMe	0%^b^
**14b**	2		2,6-lutidine	72%
**14c**	3	1.06	LiOMe	0%^c^
**14c**	3		2,6-lutidine	0%

^a^Cyclic voltammetry data were obtained relative to a Ag/AgCl reference electrode with a sweep rate of 50 mV/s. ^b^87% of the ring opened methyl ester **16b** was obtained. ^c^30% of the ring opened methyl ester **16c** was obtained.

The oxidation of **14b** to afford the six-membered ring product also proceeded well. When 2,6-lutidine was used as the base for the reaction, a 72% isolated yield of the desired product was obtained. In this case, the use of the more nucleophilic lithium methoxide as the base led to cyclization followed by opening of the lactone ring to form methyl ester product **16b**. This product did have the olefin functionalized with an alcohol and a methoxy group indicating that the initial oxidative cyclization had occurred. In this case, the slightly higher oxidation potential measured for the substrate (+0.71 V versus Ag/AgCl) is consistent with a slightly slower cyclization reaction.

Attempts to generate seven-membered ring products using the oxidative cyclization were not successful. While 30% of a methoxide opened product **16c** could be obtained with lithium methoxide as the base, no cyclic product was observed when 2,6-lutidine was employed. The reactions were quite messy and consistent with the formation of a radical cation followed by a cyclization that was too slow to compete with decomposition. The cyclic voltammetry data obtained were consistent with this observation. The +1.06 V versus Ag/AgCl oxidation potential measured for substrate **14c** was the same as that measured for the ketene dithioacetal in the absence of a trapping group [21].

The anodic coupling of a carboxylic acid group with a vinyl sulfide and an enol ether were also examined (Table 2). As with earlier alcohol and amine based cyclizations, reactions with the vinyl sulfide coupling partner proceeded much better than did their enol ether counterparts [21–22]. In the previous cases, the argument was made that less polar radical cations underwent better trapping reactions with heteroatomic nucleophiles [23], and a similar argument can be made here.

**Table 2:** Extension to other electron-rich olefins.

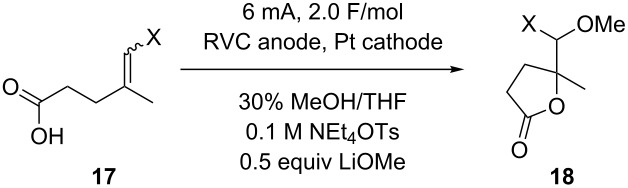		

substrate	-X	Yield

**17a**	-SMe	74%
**17b**	-OMe	66%

The oxidation potential for the vinyl sulfide used in substrate **17a** is +1.08 V versus Ag/AgCl [9] and the oxidation potential of the enol ether in substrate **17b** is +1.18 versus Ag/AgCl [9]. Both oxidation potentials are lower than the potential measured for the carboxylate suggesting that both reactions proceed through the olefinic radical cation. While an intramolecular electron-transfer to form the carboxy radical can occur, the difference in oxidation potentials suggests that the equilibrium would lie on the side of the oxidized olefin.

### Styrene substrates and additional insights

With the ketene dithioacetal, vinyl sulfide, and enol ether substrates, it appeared clear that the initial oxidation was occurring at the olefin instead of at the carboxylate. We wondered how the reaction might change if the olefin had a higher oxidation potential. With this in mind, a series of carboxylic acid substituted styrene substrates were examined (Table 3).

**Table 3:** Extension to styrene derivatives.

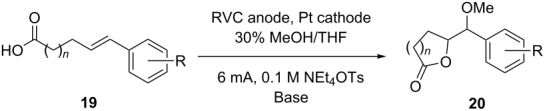						

Substrate	R	*n*	*E*_p/2_^a^	Base/Temperature	F/mol	Yield

**19a**	H	1	1.52	0.5 equiv LiOMe/rt	2	15%
**19a**	H	1	1.52	0.5 equiv LiOMe/rt	10	27%
**19a**	H	1	1.52	None/40 °C	10	33%
**19b**	H	3	1.73	NA^b^	NA	NA
**19c**	4-OMe	1	1.31	0.5 equiv LiOMe/rt	2	56%
**19c**	4-OMe	1	1.31	None/40 °C	2	76%
**19d**	4-OMe	3	1.42	NA	NA	NA
**19e**	2-OMe	1	1.39^c^	0.5 equiv LiOMe/rt	2	48%
**19e**	2-OMe	1	1.39^c^	None/40 °C	2	59%
**19f**	2-OMe	3	1.39^c^	NA	NA	NA
**19g**	3-OMe	1	1.42^c^	0.5 equiv LiOMe/rt	2	4% (NMR)
**19g**	3-OMe	1	1.42^c^	0.5 equiv LiOMe/rt	10	35%
**19g**	3-OMe	1	1.42^c^	None/40 **°**C	10	23%
**19h**	3-OMe	3	1.50	NA	NA	NA
**19i**	2,4-OMe	1	1.09	0.5 equiv LiOMe/rt	2	48%
**19i**	2,4-OMe	1	1.09	None/40 **°**C	2	45%
**19i**	2,4-OMe	1	1.09	1.0 equiv LiOMe/rt	2	74%
**19j**	2,4-OMe	3	1.11	NA	NA	NA

^a^Conditions: Substrates were dissolved in acetonitrile to a concentration of 0.025 M in a solution that contained 0.1 M tetraethylammonium tosylate. Cyclic voltammetry was performed at a sweep rate of 50 mV/s using a platinum anode. Half-wave oxidation potentials were measured versus a Ag/AgCl reference electrode. ^b^Not applicable. ^c^Lithium perchlorate was used as the electrolyte.

The first substrate studied was the simple styrene derivative **19a** (R = H). Cyclic voltammetry suggested that in the presence of base the initial oxidation should occur at the carboxylate (*E*_p/2_ = +1.38 in acetonitrile) since the *E*_p/2_ value measured for substrate **19a** under neutral conditions was +1.52 V versus Ag/AgCl. Several sets of conditions were attempted to achieve the cyclization. However, in no case was the yield of cyclic product obtained more than 33%. Proton NMR data taken of the crude reaction mixture showed no evidence for decarboxylation. Instead, all products appeared to arise from a styrene derived radical cation, an intermediate that might be formed by an intramolecular electron-transfer. Furthermore, the recovery of starting material after 2 F/mol of current had been passed through the reaction mixture indicated poor current efficiency. Why might an increase in the oxidation potential of the olefin hurt the reaction?

Past experience suggests that in such cases the low yield of product obtained might arise from either a slow initial cyclization or the formation of a stable cyclic radical that then struggles to undergo the second oxidation. Both pathways might lead to the formation of a polymer or decomposition, and in so doing lower the mass balance for the reaction as observed. Also, diffusion of a stable radical or radical cation to the cathode would result in a reduction to give the starting material, a scenario that would lead to low current efficiency. Of the two mechanistic explanations for these observations, we believe that a slow second oxidation step is the problem.

The possibility of a slow cyclization was eliminated by consideration of the observed half-wave oxidation potential measured for **19a**. The oxidation potential (*E*_p/2_) of +1.52 V for **19a** (measured under neutral conditions) was significantly lower than that measured for **19b** (+1.73 V), a similar styrene with a longer tether between the coupling partners. This observation is consistent with the oxidation of **19a** leading to a fast cyclization that rapidly removes the radical cation from the electrode surface, resulting in a lower observed oxidation potential [21]. The seven-membered ring cyclization arising from the oxidation of **19b** would be significantly slower, giving rise to a higher oxidation potential.

**Scheme 5:**
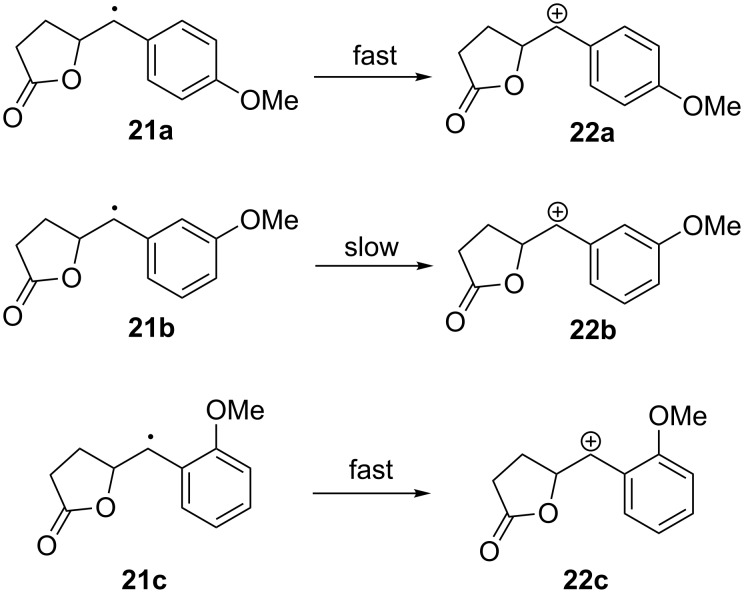
Predicted relative rates of single-electron oxidation based on resonance stabilization of the resulting cation.

Given the evidence for a fast cyclization reaction, we attributed the challenges associated with the styrene cyclizations to a problem with the second single-electron oxidation. This idea was supported by our next set of experiments. Methoxy-substituted styrenes **19c** (R = 4-OMe), **19e** (R = 2-OMe), and **19g** (R = 3-OMe) were prepared. Substrate **19c** has a *p-*methoxy substituent positioned perfectly for aiding in the removal of a second electron from cyclic radical **21a** (Scheme 5). Indeed, the reactions of **19c** proceeded better than the cyclizations originating from **19a**, giving acceptable yields and complete consumption of the starting material after 2 F/mol of current had been passed. In this case, the reaction benefited strongly from using less basic reaction conditions and slightly elevated temperatures. When more basic reaction conditions were used, an overoxidized product derived from the elimination of the lactone ester to form a methoxy styrene from product **20c** was obtained. The elevated temperatures are known to assist the intramolecular cyclization. The significantly lower oxidation potential measured for **19c** relative to that measured for its seven-membered ring counterpart **19d** was again consistent with a fast initial cyclization.

We believe that the primary advantage of the methoxy group is to facilitate the second electron oxidation, rather than merely stabilizing an olefinic radical cation. To demonstrate this, the position of the methoxy group on the ring was altered systematically (see Table 3). Cyclic radical **21b**, arising from the reaction of *m*-methoxy substituted styrene **19g** (Table 3), would not be expected to oxidize as readily as radical **21a**. Indeed, the anodic oxidation of **19g** gave low yields and poor current efficiency, mirroring the results obtained from the oxidation of unsubstituted styrene **19a**. As with other substrates, the oxidation potential of **19g** was lower than that of its seven-membered ring counterpart **19h**, indicating a fast cyclization. Hence, the poor yield obtained from the oxidation of **19g** was not due to a problem with the first step.

**Figure 1:**
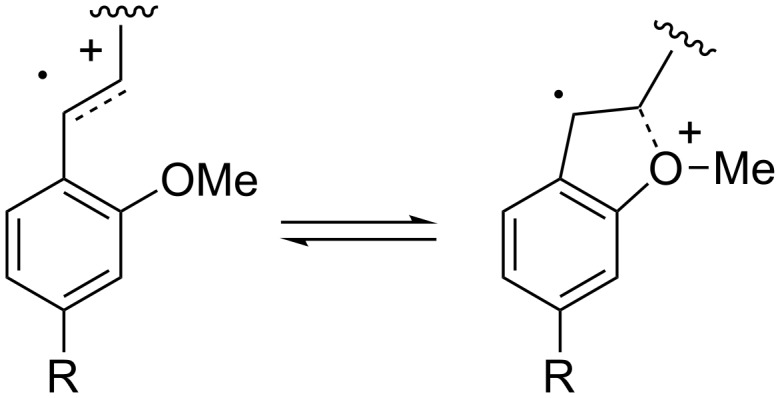
Radical cation stabilization by an *o*-methoxy substituent.

Good yields and efficient consumption of starting material were regained upon moving the methoxy group to the *ortho* position (**19e**). Interestingly, **19e** showed no oxidation potential drop from the analogous long-chain substrate **19f**. It is tempting to assign this to a slow cyclization, but we do not believe that this is necessarily the case. The relatively low oxidation potentials for **19e** and **19f** (as well as the potentials measured for **19i** and **19j**) may be due to an interaction between the *o*-methoxy substituent and the radical cation (Figure 1). Such a stabilizing interaction would be expected to lower the oxidation potential of the olefin.

We also investigated the reactivity of the *o*,*p*-dimethoxy substituted styrene **19i** towards the coupling reaction. This reaction also led to a good yield of product and efficient consumption of the starting material. Surprisingly, the optimized conditions for the oxidation of **19i** were different from those optimized for the oxidation of either **19c** or **19e**, and the initial yields for the cyclization of **19i** were not good. The cyclization originating from **19i** benefited from the use of more base. It appears that the use of base reduced the formation of a quinone methide from the product. For example, when a full equivalent of base was used for the reaction, the desired product was obtained in 74% isolated yield.

It is clear that the success of the styrene derived coupling reaction depends strongly on the position of the methoxy group on the aromatic ring. *Ortho* and *para* substitution leads to a successful cyclization while *meta* substitution does not. This observation is best explained by the reaction being dependent upon the ease with which the cyclic benzyl radicals **21a–c** are oxidized.

## Conclusion

It was found that carboxylic acid nucleophiles can be coupled to electron-rich olefins to form lactone products in good yield without interference from competitive decarboxylation reactions. The reactions are consistent with carboxylate trapping of an olefin-derived radical cation intermediate and depend strongly on the ease with which the second oxidation step in the mechanism occurs. This is particularly true when styrene-based substrates are used. Finally, optimization of reaction pH required careful consideration of product stability.

## Supporting Information

File 1 Procedures for electrolysis and cyclic voltammetry experiments, characterization of electrolysis products, procedures for synthesis and characterization of electrolysis starting materials.
