# Supplementary material for: Anodic coupling of carboxylic acids to electron-rich double bonds: A surprising non-Kolbe pathway to lactones
Source: Beilstein J Org Chem. 2013 Aug 9;9:1630–6. doi: 10.3762/bjoc.9.186 (PMC3778411; doi:10.3762/bjoc.9.186)
Supplement: File 1 — Procedures for electrolysis and cyclic voltammetry experiments, characterization of electrolysis products, procedures for synthesis and characterization of electrolysis starting materials. [file Beilstein_J_Org_Chem-09-1630-s001.pdf]

Supporting Information

for

Anodic coupling of carboxylic acids to electron-rich double bonds: A surprising non-Kolbe pathway to lactones

Robert J. Perkins, Hai-Chao Xu, John M. Campbell and Kevin D. Moeller*

Address: Washington University in Saint Louis, Saint Louis, Missouri 63130, United States

Email: Kevin D. Moeller - moeller@wustl.edu

* Corresponding author

Procedures for electrolysis and cyclic voltammetry experiments, characterization of electrolysis products, procedures for synthesis and characterization of electrolysis starting materials

Contents

1 Electrolysis Experiments S2

2 Cyclic Voltammetry Experiments S2

3 Characterization of Electrolysis Products S2

4 Substrate Synthesis and Characterization S6

5 NMR Spectra S13

1 Electrolysis Experiments

Lithium methoxide (1.0 M in methanol, 0 to 1.0 equivalents, see text) was added to either a methanol or 30% methanol / tetrahydrofuran solution of substrate (0.03 M, 1 equivalent) and the electrolyte tetraethylammonium *p*-toluenesulfonate in a three-neck round bottom flask at room temperature under argon atmosphere. Two of the three septa were replaced by a reticulated vitreous carbon anode (100 PPI) and a platinum wire cathode. The electrolysis was performed at a constant current of 6 mA.

When complete, the reaction was concentrated *in vacuo*, then water and diethyl ether were added. The water layer was separated and extracted three times with ether and the organic washes combined, dried over sodium sulfate, and concentrated *in vacuo*. The residue was then chromatographed through a silica gel column (slurry packed with 1% triethylamine using in ether/hexane eluent) to give the desired product.

Alternatively, once the reaction is complete, the solvent may be removed in vacuo to give a crude residue deposited onto the electrolyte tetraethylammonium *p*-toluenesulfonate. This may then be chromatographed in the same manner as described above.

2 Cyclic Voltammetry Experiments

A solution of the substrate (0.025 M) and tetraethylammonium tosylate (0.1 M) in the necessary solvent (noted in the text) was prepared. Cyclic voltammetry was performed with a sweep rate of 50 mV/s, with the sweep beginning in the positive direction. The half-wave oxidation potentials ($E_{p/2}$) were measured from the resulting oxidation curve.

3 Characterization of Electrolysis Products

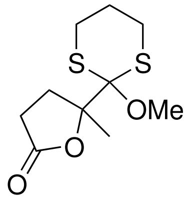

5-(2-methoxy-1,3-dithian-2-yl)-5-methyldihydrofuran-2(3H)-one, 15a

Spectral data for the title compound matched that which has been previously reported (Liu, B.; Duan, S.; Sutterer, A. C.; Moeller, K. D. *J. Am. Chem. Soc.*, **2002**, *124*, 10101-10111).

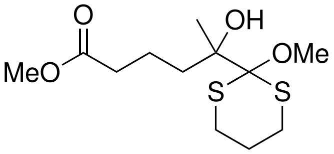

methyl 5-hydroxy-5-(2-methoxy-1,3-dithian-2-yl)hexanoate, 16b

IR (neat, cm^{-1}) 3499, 1736, 1087.

^{1}H-NMR (300 MHz, $CDCl_3$) δ 3.64 (s, 3H), 3.54 (s, 3H), 2.96-2.80 (m, 4H), 2.34-2.29 (m, 3H), 1.97-1.70 (m, 6H), 1.35 (s, 3H).

^{13}C-NMR (75 MHz, $CDCl_3$) δ 174.3, 104.2, 81.4, 53.1, 51.7, 35.7, 34.6, 27.9, 27.9, 22.4, 21.6, 19.6.

ESI HRMS *m/z* $(M+Na)^+$ calculated 317.0852, observed 317.0860.

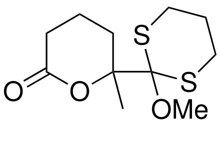

6-(2-methoxy-1,3-dithian-2-yl)-6-methyltetrahydro-2H-pyran-2-one, 15b
IR (neat, cm^{-1}) 1738, 1255, 1089.
^{1}H-NMR (300 MHz, $CDCl_3$) δ 3.58 (s, 3H), 3.05-2.84 (m, 4H), 2.63-2.54 (m, 1H), 2.45-2.37 (m, 1H), 2.27-2.16 (m, 1H), 2.00-1.73 (m, 5H), 1.58 (m, 3H).
^{13}C-NMR (75 MHz, $CDCl_3$) δ 170.9, 102.8, 90.9, 53.1, 30.4, 29.4, 27.9, 22.7, 22.6, 17.4.
ESI HRMS m/z $(M+H)^+$ calculated 263.0770, observed 263.0771.

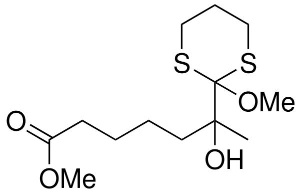

methyl 6-hydroxy-6-(2-methoxy-1,3-dithian-2-yl)heptanoate, 16c
IR (neat, cm^{-1}) 3480, 1735, 1680, 1173.
^{1}H-NMR (300 MHz, $CDCl_3$) δ 3.68 (s, 3H), 3.59 (s, 3H), 3.02-2.84 (m, 4H), 2.36 (t, J = 7.5 Hz, 2H), 2.21 (s, 1H), 2.04-1.40 (m, 8H), 1.37 (s, 3H).
^{13}C-NMR (75 MHz, $CDCl_3$) δ 174.4, 104.4, 81.5, 53.1, 51.7, 36.0, 34.4, 28.0, 25.7, 23.6, 22.4, 21.6.
ESI HRMS m/z $(M\text{-}OCH_3)^+$ calculated 277.0932, observed 277.0897.

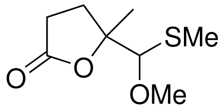

5-(methoxy(methylthio)methyl)-5-methyldihydrofuran-2(3H)-one, 18a
IR (neat, cm^{-1}) 1772, 1190, 1094.
^{1}H-NMR (300 MHz, $CDCl_3$) δ 4.21 (s, 0.5 H), 4.12 (s, 0.5 H), 3.47 (s, 1.5 H), 3.45 (s, 1.5 H), 2.78-2.21 (m, 3H), 2.17, 2.13 (2s, 3H), 1.51, 1.50 (2s, 3H).
^{13}C-NMR (75 MHz, $CDCl_3$) δ 77.2, 177.1, 95.2, 94.9, 88.9, 88.6, 59.1, 57.5, 30.8, 29.9, 29.7, 29.6, 25.0, 14.7, 14.0.
ESI HRMS m/z $(M+H)^+$ calculated 191.0731, observed 191.0736.

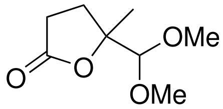

5-(dimethoxymethyl)-5-methyldihydrofuran-2(3H)-one, 18b
IR (neat, cm^{-1}) 1776, 1104, 1078.
^{1}H-NMR (300 MHz, $CDCl_3$) δ 4.13 (s, 1H), 3.50 (s, 3H), 3.48 (s, 3H), 2.71-2.51 (m, 1H), 2.50-2.38 (m, 2H), 1.82-1.71 (m, 1H), 1.37 (s, 3H).
^{13}C-NMR (75 MHz, $CDCl_3$) δ 177.6, 109.5, 86.9, 58.7, 57.5, 29.7, 28.3, 23.3.
ESI HRMS m/z $(M+H)^+$ calculated 175.0965, observed 175.0966.

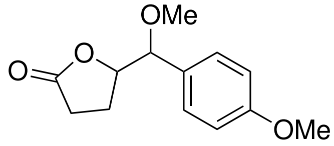

5-[methoxy(4-methoxyphenyl)methyl]dihydrofuran-2(3H)-one, 20c
Isolated as a 2:1 mixture of diastereomers

IR (neat, cm^{-1}) 2937, 1778, 1611, 1584, 1513.

^{1}H-NMR (300 MHz, $CDCl_3$) δ 7.30-7.21 (m, 2H), 6.96-6.87 (m, 2H), 4.65 (dt, Jd = 5.7, Jt = 7.0 Hz, 0.7H), 4.57 (ddd, J = 8.4, 4.8, 3.6 Hz, 0.3H), 4.41 (d, J = 3.6 Hz, 0.3H), 4.20 (d, J = 5.7 Hz, 0.7H), 3.82 (s, 3H), 3.30 (s, 1H), 3.28 (s, 2H), 2.59-2.19 (m, 2H), 2.12-1.92 (m, 2H)

^{13}C-NMR (75 MHz, $CDCl_3$) δ 177.7, 177.3, 160.0, 159.7, 128.9, 128.4, 128.3, 114.3, 114.2, 84.7, 83.9, 82.9, 82.2, 57.6, 57.0, 55.4, 28.5, 28.3, 24.2, 21.7.

ESI HRMS m/z $(M+H)^+$ calculated 237.1121, observed 237.1122.

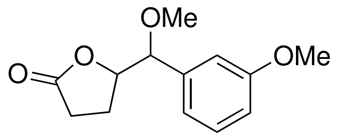

5-[methoxy(3-methoxyphenyl)methyl]dihydrofuran-2(3H)-one, 20g

Isolated as two separate diastereomers in a 2:1 ratio.

Major diastereomer:

IR (neat, cm^{-1}) 2938, 2360, 2341, 1775, 1600, 1585.

^{1}H-NMR (300 MHz, $CDCl_3$) δ 7.34-7.25 (m, 1H), 6.96-6.83 (m, 3H), 4.66 (dt, Jd = 6.0 Hz, Jt = 6.9 Hz, 1H), 4.22 (d, J = 5.7 Hz, 1H), 3.82 (s, 3H), 3.31 (s, 3H), 2.46-2.15 (m, 2H), 2.07-1.93 (m, 2H).

^{13}C-NMR (75 MHz, $CDCl_3$) δ 177.0, 159.9, 138.0, 129.7, 119.9, 114.0, 113.0, 85.0, 81.9, 57.2, 55.3, 28.2, 24.1.

ESI HRMS m/z $(M+H)^+$ calculated 237.1121, observed 237.1122.

Minor diastereomer:

IR (neat, cm^{-1}) 2938, 2360, 2341, 1777, 1601, 1586.

^{1}H-NMR (300 MHz, $CDCl_3$) δ 7.34-7.27 (m, 1H), 6.97-6.81 (m, 3H), 4.60 (ddd, J = 8.1, 4.8, 3.0 Hz, 1H), 4.46 (d, J = 3.3 Hz), 3.83 (s, 3H), 3.34 (s, 3H), 2.64-2.21 (m, 3H), 2.12-1.94 (m, 1H).

^{13}C-NMR (75 MHz, $CDCl_3$) δ 177.6, 160.0, 138.4, 129.8, 119.2, 113.5, 112.4, 84.0, 82.6, 55.8, 55.3, 28.4, 21.4.

ESI HRMS m/z $(M+H)^+$ calculated 237.1121, observed 237.1122.

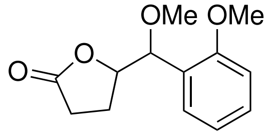

5-[methoxy(2-methoxyphenyl)methyl]dihydrofuran-2(3H)-one, 20e

Isolated as two separate diastereomers in a 2:1 ratio.

Major diastereomer

IR (neat, cm^{-1}) 2938, 1776, 1600, 1588.

^{1}H-NMR (300 MHz, $CDCl_3$) δ 7.39 (dd, J = 7.4, 1.5 Hz, 1H), 7.30 (ddd, J = 8.4, 7.4, 2.1 Hz, 1H), 7.00 (td, Jt = 7.5 Hz, Jd = 0.6 Hz, 1H), 6.90 (d, J = 8.4 Hz, 1H), 4.75-4.62 (m, 2H), 3.84 (s, 3H), 3.28 (s, 3H), 2.65-2.50 (m, 1H), 2.48-2.34 (m, 1H), 2.18-1.97 (m, 2H).

^{13}C-NMR (75 MHz, $CDCl_3$) δ 177.7, 157.4, 129.4, 128.2, 125.2, 121.2, 110.6, 82.4, 78.9, 57.5, 55.6, 28.7, 24.3.

ESI HRMS m/z $(M+H)^+$ calculated 237.1121, observed 237.1122.

Minor diastereomer

IR (neat, cm^{-1}) 2939, 2360, 2342, 1776, 1601, 1588.

^{1}H-NMR (300 MHz, $CDCl_3$) δ 7.38 (dd, J = 7.5, 1.8 Hz, 1H), 7.34-7.27 (m, 1H), 7.01 (t, J = 7.5 Hz, 1H), 6.89 (d, J = 8.1 Hz, 1H), 4.94 (d, J = 2.4 Hz, 1H), 4.71 (ddd, J = 8.4, 4.5, 2.4 Hz, 1H), 3.84 (s, 3H), 3.33 (s, 3H), 2.63 (ddd, J = 17.7, 9.6, 8.0 Hz, 1H), 2.43-2.16 (m, 2H), 1.96-1.80 (m, 1H).

^{13}C-NMR (75 MHz, $CDCl_3$) δ 178.1, 156.8, 129.0, 126.8, 124.6, 120.7, 110.2, 80.8, 78.6, 57.9, 55.2, 28.6, 20.8.

ESI HRMS m/z $(M+H)^+$ calculated 237.1121, observed 237.1122.

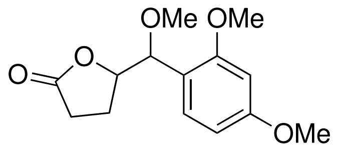

5-[(2,4-dimethoxyphenyl)(methoxy)methyl]dihydrofuran-2(3H)-one, 20i

Isolated as two separate diastereomers in a 2:1 ratio.

Major diasteriomer

IR (neat, cm^{-1}) 939, 2359, 1776, 1611, 1588, 1506.

^{1}H-NMR (300 MHz, $CDCl_3$) δ 7.28 (d, J = 8.4 Hz, 1H), 6.53 (dd, J = 8.4, 2.4 Hz, 1H), 6.47 (d, 2.4 Hz, 1H), 4.72-4.57 (m, 2H), 3.82 (s, 3H), 3.81 (s, 3H), 2.64-2.32 (m, 2H), 2.14-1.96 (m, 2H).

^{13}C-NMR (75 MHz, $CDCl_3$) δ 177.6, 160.9, 158.5, 128.9, 117.4, 104.5, 98.4, 82.7, 78.8, 57.1, 55.53, 55.51, 28.6, 24.2.

ESI HRMS m/z $(M+Na)^+$ calculated 289.1046, observed 289.1045.

Minor diasteriomer

IR (neat, cm^{-1}) 2939, 1775, 1612, 1588, 1505.

^{1}H-NMR (300 MHz, $CDCl_3$) δ 7.27 (d, J = 8.4 Hz, 1H), 6.53 (dd, J = 8.4, 2.4 Hz, 1H), 6.46 (d, J = 2.4 Hz, 1H), 4.85, (d, J = 2.7 Hz, 1H), 4.71-4.62 (m, 1H), 3.82 (s, 3H), 3.81 (s, 3H), 3.31 (s, 3H), 2.60 (ddd, J = 17.7, 10.2, 7.8 Hz, 1H), 2.43-2.16 (m, 2H), 1.99-1.82 (m, 1H).

^{13}C-NMR (75 MHz, $CDCl_3$) δ 178.3, 160.9, 158.1, 127.9, 117.0, 104.5, 98.6, 81.3, 78.6, 57.9, 55.6, 55.5, 28.8, 21.1.

ESI HRMS m/z $(M+Na)^+$ calculated 289.1046, observed 289.1046.

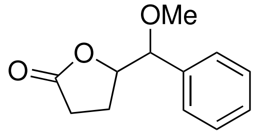

5-[methoxy(phenyl)methyl]dihydrofuran-2(3H)-one, 20a

Isolated as two separate diastereomers in a 1:1 ratio.

First diastereomer

IR (neat, cm^{-1}) 2933, 2360, 2341, 1777.

^{1}H-NMR (300 MHz, $CDCl_3$) δ 7.45-7.28 (m, 5H), 4.59 (ddd, J = 7.8, 4.8, 3.3 Hz, 1H), 4.48 (d, 3.3 Hz, 1H), 3.33 (s, 3H), 2.64-2.46 (m, 1H), 2.45-2.21 (m, 2H), 2.09-1.93 (m, 1H).

^{13}C-NMR (75 MHz, $CDCl_3$) δ 177.6, 136.7, 128.7, 128.3, 126.9, 84.1, 82.7, 57.7, 28.4, 21.4.

ESI HRMS m/z $(M+H)^+$ calculated 207.1016, observed 207.1016.

Second diastereomer

IR (neat, cm^{-1})

^{1}H-NMR (300 MHz, $CDCl_3$) δ 7.49-7.28 (m, 5H), 4.67 (dt, Jd = 5.7 Hz, Jt = 6.9 Hz, 1H), 4.25 (d, J = 5.7 Hz), 3.31 (s, 3H), 2.46-2.15 (m, 2H), 2.05-1.94 (m, 2H).

^{13}C-NMR (75 MHz, $CDCl_3$) δ 177.0, 136.4, 128.7, 128.6, 127.6, 85.0, 81.9, 57.2, 28.2, 24.0.

ESI HRMS m/z $(M+H)^+$ calculated 207.1016, observed 207.1016.

4 Substrate Synthesis and Characterization

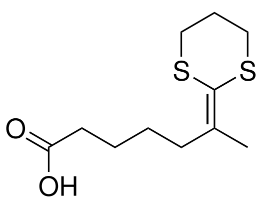

6-(1,3-dithian-2-ylidene)heptanoic acid, 14c

2-(Trimethylsilyl)-1,3-dithiane (1.467g, 7.629 mmol, 2 equivalents) was dissolved in tetrahydrofuran (10 mL) under inert atmosphere, cooled to -78 °C, and treated with *n*-butyllithium (1.6 M in hexanes, 4.77 mL, 7.629 mmol, 2 equivalents). The resulting solution was stirred at -78 °C for 30 minutes, then at room temperature for 30 minutes. The solution was then cooled back to -78 °C and treated with 6-oxoheptanoic acid (0.6070g, 3.789 mmol, 1 equivalent, dissolved in 10 mL of tetrahydrofuran). After 15 minutes, cold bath was removed and the reaction was allowed to warm to room temperature as it stirred overnight. The reaction was quenched the following morning with 3 M aqueous hydrochloric acid to a pH of approximately 6. Ether was added, and the layers were separated. The aqueous layer was extracted twice with dichloromethane. The combined organic extracts were dried over magnesium sulfate, filtered, and concentrated in vacuo. The crude residue was chromatographed through silica gel with hexanes and ethyl acetate to give the desired product (67%) as a colorless oil.

IR (neat, cm^{-1}) 1706, 1420, 1276.

^{1}H-NMR (300 MHz, $CDCl_3$) δ 2.97-2.91 (m, 4H), 2.50-2.44 (m, 4H), 2.24-2.16 (m, 2H), 1.99 (s, 3H), 1.77-1.67 (m, 2H), 1.57-1.51 (m, 2H).

^{13}C-NMR (75 MHz, $CDCl_3$) δ 180.6, 140.3, 120.0, 35.6, 34.3, 30.6, 30.5, 27.6, 25.3, 24.6, 20.5.

ESI HRMS *m/z* $(M+H)^+$ calculated 247.0821, observed 247.0821.

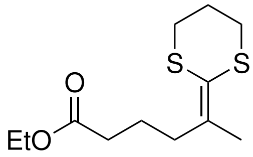

ethyl 5-(1,3-dithian-2-ylidene) hexanoate, S-1

The title compound was prepared according to the same procedure for the synthesis of **S-2**, using ethyl 5-oxohexanoate instead of ethyl levulinate. Following that procedure, the title compound was prepared in a 75% yield.

IR (neat, cm^{-1}) 1732, 1244, 1147.

^{1}H-NMR (300 MHz, $CDCl_3$) δ 4.12 (q, J = 6.9 Hz, 2H), 2.88-2.81 (m, 4H), 2.38 (t, J = 7.5 Hz, 2H), 2.28 (t, J = 7.5 Hz, 2H), 2.14-2.06 (m, 2H), 1.89 (s, 3H), 1.78-1.67 (m, 2H), 1.25 (t, J = 6.9 Hz, 3H).

^{13}C-NMR (75 MHz, $CDCl_3$) δ 173.5, 139.0, 120.7, 60.3, 31.2, 33.9, 30.4, 30.2, 25.1, 23.2, 14.4.

ESI HRMS *m/z* $(M+H)^+$ calculated 261.0977, observed 261.0978.

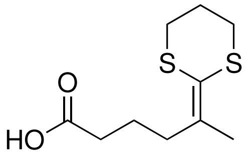

5-(1,3-dithian-2-ylidene)hexanoic acid, 14b

To a solution of **S-1** (1.32g, 5.07 mmol) in tetrahydrofuran (30 mL) and water (10 mL) was added lithium hydroxide monohydrate (1.06 g, 25.2 mmol). The reaction mixture was stirred overnight. 1 N

aqueous hydrochloric acid was added to adjust the pH of the aqueous solution to 2. Ether was then added and organic phase separated. The aqueous phase was exacted with ether twice. The organic phase was combined, dried over magnesium sulfate, filtered, and concentrated under vacuum to give 9.2c (0.78 g, 67%) as a white solid.

IR (neat, cm^{-1}) 3093, 2034, 2908, 2848, 2662, 1706, 1420.

^{1}H-NMR (300 MHz, $CDCl_3$) δ 2.89-2.82 (m, 4H), 2.43-2.33 (m, 4H), 2.14-2.06 (m, 2H), 1.90 (s, 3H), 1.80-1.70 (m, 2H).

^{13}C-NMR (75 MHz, $CDCl_3$) δ 179.7, 138.9, 120.9, 35.1, 30.5, 30.4, 30.2, 25.1, 22.9, 20.2.

ESI HRMS m/z $(M+H)^+$ calculated 233.0664, observed 233.0665.

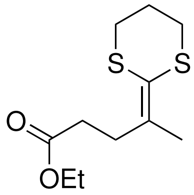

Ethyl 4-(1,3-dithian-2-ylidene) pentanoate, S-2

The preparation and characterization of the title compound from **S-2** was performed using according to a previously reported procedure (Liu, B.; Duan, S.; Sutterer, A. C.; Moeller, K. D. *J. Am. Chem. Soc.*, **2002**, *124*, 10101-10111).

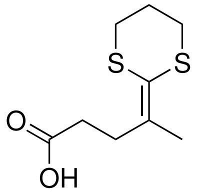

4-(1,3-Dithian-2-ylidene)pentanoic acid, 14a

The preparation and characterization of the title compound from **S-2** was performed using according to a previously reported procedure (Liu, B.; Duan, S.; Sutterer, A. C.; Moeller, K. D. *J. Am. Chem. Soc.*, **2002**, *124*, 10101-10111).

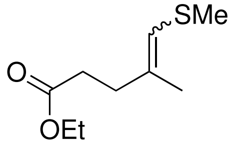

ethyl 4-methyl-5-(methylthio)pent-4-enoate, S-3

To a suspension of (methylthiomethyl)triphenylphosphonium chloride (10.7 g, 30 mmol) in tetrahydrofuran (100mL) was added an *n*-butyllithium solution (1.6 M in hexanes, 18.7 mL, 30 mmol) at 0 °C under argon atmosphere. After the addition was complete, the clear solution was stirred at 0 °C for 30 min and then treated with ethyl levulinate (4.2 mL, 30 mmol). The reaction was warmed to room temperature and then stirred overnight. The reaction was cooled to 0 °C and brine and ether were added. The organic phase was separated and the aqueous layer extracted with ether. The combined organic layers were dried over magnesium sulfate and concentrated. Chromatography on silica gel gave the desired product as a colorless oil (2.5 g, 45%).

IR (neat, cm^{-1}) 1732, 1372, 1157.

^{1}H-NMR (300 MHz, $CDCl_3$) δ 5.62 (s, 1H), 4.09 (q, J = 7.2 Hz, 2H), 2.44-2.32 (m, 4H), 2.21 (s, 3H), 1.69 (m, 3H), 1.22 (t, J = 7.2 Hz, 3H).

^{13}C-NMR (75 MHz, $CDCl_3$) δ 173.2, 134.1, 121.8, 60.5, 34.4, 33.2, 17.8, 17.3, 14.4.

ESI HRMS m/z $(M+H)^+$ calculated 189.0944, observed 189.0944.

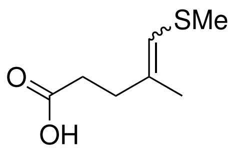

4-methyl-5-(methylthio)pent-4-enoic acid, 17a

The title compound was prepared from ethyl 4-methyl-5-(methylthio)pent-4-enoate (**S-3**) using the same procedure for the synthesis of 4-(1,3-Dithian-2-ylidene)pentanoic acid, **14a**.

IR (neat, cm^{-1}) 1710, 1436, 1301.

^{1}H-NMR (300 MHz, $CDCl_3$) δ 11.8 (br, 1H), 5.65 (s, with fine couplings, 1H), 2.49-2.34 (m, 4H), 2.22, 2.21 (2s, 3H), 1.75 (d, J = 1.5 Hz, 1.5 H), 1.70 (s, 1.5 H).

^{13}C-NMR (75 MHz, $CDCl_3$) δ 180.0, 179.9, 134.5, 133.6, 122.5, 122.1, 34.0, 33.0, 32.0, 28.8, 22.9, 17.9, 17.5, 17.3.

ESI HRMS m/z $(M+H)^+$ calculated 161.0631, observed 161.0631.

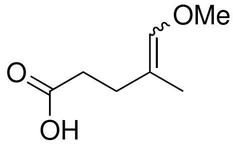

5-methoxy-4-methyl pent-4-enoic acid, 17b

To a suspension of (methoxymethyl)triphenylphosphonium chloride (3.693 g, 10.77 mmol, 2.5 eq) in THF (15 mL) was added sodium bis(trimethylsilyl) amide (1.0 M in tetrahydrofuran, 10.8 mL, 2.5 eq) at 0°C under argon atmosphere. The resulting solution was stirred at 0°C for 30 min. Levulinic acid (0.5 g, 4.306 mmol, 1 eq) was then added via 1 mL tetrahydrofuran. The resulting suspension was allowed to stir overnight and warm to room temperature. After 22 hours, water was added and the Aqueous layer was washed with ether three times. The aqueous layer was then acidified with hydrochloric acid (3 M, aq) to a pH of 5. The aqueous layer was then extracted three times with diethyl ether. The combined organic extracts were dried over magnesium sulfate, concentrated in vacuo, and chromatographed through silica gel with a mixture of hexanes and ethyl acetate to give the product as a colorless oil and a 2:1 mixture of isomers. (0.2434 g, 39%).

IR (neat, cm^{-1}) 2933, 2857, 2873, 2741, 2674, 1709.

^{1}H-NMR (600 MHz, $CDCl_3$) δ 12/03 (bs, 1H), 5.83 (s, 0.33H), 5.77 (s, 0.66H), 3.54 (s, 1H), 3.51 (s, 2H), 2.47-2.40 (m, 2H), 2.40-2.35 (m, 1.33H), 2.21 (t, J = 7.2 Hz, 0.66H), 1.59 (s, 1H), 1.54 (s, 2H).

^{13}C-NMR (150 MHz, $CDCl_3$) δ 180.4, 180.0, 142.9, 142.8, 112.0, 111.8, 60.6, 59.3, 33.3, 32.5, 29.2, 24.6, 17.2, 12.5.

ESI HRMS m/z $(M+H)^+$ calculated 145.0859, observed 145.0859.

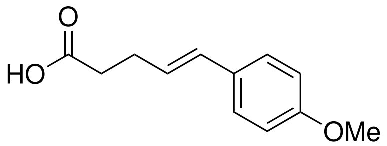

(4E)-5-(4-methoxyphenyl)pent-4-enoic acid, 19c

To a solution of (3-carboxypropyl)triphenylphosphonium bromide (1.1 g, 2.5 mmol, 1.2 eq.) in tetrahydrofuran (10 ml) was added dropwise a solution of sodium bis(trimethylsilyl)amide (1.0 M in tetrahydrofuran, 5.0 ml, 5.0 mmol, 2.4 eq.) at 0 °C. The solution was stirred for 30 min, then cooled to -78 °C. P-anisaldehyde (0.25 ml, 2.0 mmol, 1 eq.) was then added dropwise. The reaction was allowed to warm to room temperature overnight. Water and ether were added. The water layer was separated and acidified with 1 M aqueous

hydrochloric acid to pH = 1, then extracted twice with ethyl acetate. The combined organic layers were dried over sodium sulfate, filtered, and concentrated in vacuo. The crude product was chromatographed on silica gel (1:1 to 5:1 ether:hexanes) to give 0.31 g of product (73%) as a white solid.

IR (neat, cm^{-1}) 2932, 2358, 2340, 1694, 1606.

^{1}H-NMR (300 MHz, $CDCl_3$) δ 7.27 (d, J = 8.7 Hz, 2H), 6.84 (d, J = 8.7 Hz, 2H), 6.39 (d, J = 15.9 Hz, 1H), 6.13-6.00 (m, 1H), 3.80 (s, 3H), 2.55-2.50 (m, 4H).

^{13}C-NMR (75 MHz, $CDCl_3$) δ 179.5, 159.1, 130.8, 130.3, 127.4, 126.0, 114.1, 55.5, 34.1, 28.1.

ESI HRMS m/z $(M+H)^+$ calculated 207.1016, observed 207.1016.

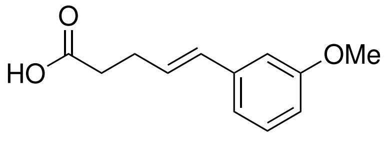

(4E)-5-(3-methoxyphenyl)pent-4-enoic acid, 19g

To a solution of (3-carboxypropyl)triphenylphosphonium bromide (2.0 g, 4.8 mmol, 1.1 eq.) in tetrahydrofuran (20 ml) was added dropwise a solution of lithium bis(trimethylsilyl)amide (1.0 M in tetrahydrofuran, 9.5 ml, 9.5 mmol, 2.2 eq.) at 0 °C. The solution was stirred for 30 min, then cooled to -78 °C. M-anisaldehyde (0.54 ml, 4.4 mmol, 1 eq.) was then added dropwise. The reaction was allowed to warm to room temperature overnight. Water and ether were added. The water layer was separated and acidified with 1 M aqueous hydrochloric acid to pH = 1, then extracted twice with ethyl acetate. The combined organic layers were dried over sodium sulfate, filtered, and concentrated in vacuo. The crude product was chromatographed on silica gel (3:1 ether:hexanes) to give 0.83 g of product (91%) as a white solid.

IR (neat, cm^{-1}) 3026, 2360, 2342, 1707, 1598, 1578.

^{1}H-NMR (300 MHz, $CDCl_3$) δ 7.21 (t, J = 7.9 Hz, 1H), 6.94 (d, J = 7.9 Hz, 1H), 6.88 (s, with fine couplings, 1H), 6.83-6.75 (m, 1H), 6.43 (d, J = 15.6 Hz, 1H), 6.28-6.15 (m, 1H), 3.81 (s, 3H), 2.57-2.53 (m, 4H).

^{13}C-NMR (75 MHz, $CDCl_3$) δ 179.6, 159.8, 138.8, 131.1, 129.5, 128.4, 118.8, 112.9, 111.6, 55.2, 33.8, 27.9.

ESI HRMS m/z $(M+Na)^+$ calculated 229.0835, observed 229.0835.

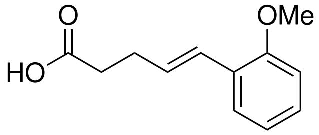

(4E)-5-(2-methoxyphenyl)pent-4-enoic acid, 19e

To a solution of (3-carboxypropyl)triphenylphosphonium bromide (2.0 g, 4.8 mmol, 1.1 eq.) in tetrahydrofuran (20 ml) was added dropwise a solution of sodium bis(trimethylsilyl)amide (1.0 M in THF, 9.5 ml, 9.5 mmol, 2.2 eq.) at 0 °C. The solution was stirred for 30 min, then cooled to -78 °C. A solution of o-anisaldehyde (0.60 g, 4.4 mmol, 1 eq.) in tetrahydrofuran (2 ml) was then added dropwise. The reaction was allowed to warm to room temperature overnight. Water and ether were added. The water layer was separated and acidified with 1 M aqueous hydrochloric acid to pH = 1, then extracted twice with ethyl acetate. The combined organic layers were dried over sodium sulfate, filtered and concentrated in vacuo. The crude product was chromatographed on silica gel (2:1 ether:hexanes) to give 0.55 g of product (60%) as a white solid.

IR (neat, cm^{-1}) 3034, 2341, 2360, 1706, 1596.

^{1}H-NMR (300 MHz, $CDCl_3$) δ 7.40 (dd, J = 7.7, 1.7 Hz, 1H), 7.20 (ddd, J = 8.4, 7.7, 1.5 Hz, 1H), 6.90 (t, with fine couplings, J = 7.7 Hz, 1H), 6.85 (dd, J = 8.4, 0.9 Hz, 1H), 6.77 (d, J = 16.2 Hz, 1H), 6.27-6.13 (m, 1H), 3.84 (s, 3H), 2.59-2.54 (m, 4H).

^{13}C-NMR (75 MHz, $CDCl_3$) δ 180.0, 156.5, 128.7, 128.3, 126.7, 126.4, 126.0, 120.7, 110.9, 55.5, 34.0, 28.4.

ESI HRMS m/z $(M+Na)^+$ calculated 229.0835, observed 229.0835.

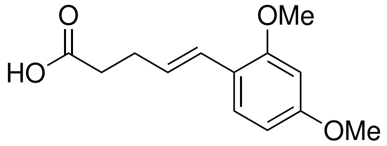

(4E)-5-(2,4-dimethoxyphenyl)pent-4-enoic acid, 19i

To a solution of (3-carboxypropyl)triphenylphosphonium bromide (2.1 g, 4.9 mmol, 1.2 eq.) in tetrahydrofuran (20 ml) was added dropwise a solution of sodium bis(trimethylsilyl)amide (1.0 M in THF, 9.7 ml, 9.7 mmol, 2.4 eq.) at 0 °C. The solution was stirred for 30 min, then cooled to -78 °C. A solution of 2,4-dimethoxybenzaldehyde (0.73 g, 4.0 mmol, 1 eq.) in tetrahydrofuran (3 ml) was then added dropwise. The reaction was allowed to warm to room temperature overnight. Water and ether were added. The water layer was separated and acidified with 1 M aqueous hydrochloric acid to pH = 1, then extracted twice with ethyl acetate. The combined organic layers were dried over sodium sulfate, filtered and concentrated in vacuo. The crude product was chromatographed on silica gel (3:1 ether:hexanes) to give 0.67 g of product (70%) as a white solid.

IR (neat, cm^{-1}) 2995, 2361, 1705, 1610, 1578, 1503.

^{1}H-NMR (300 MHz, $CDCl_3$) δ 7.31 (d, J = 8.1 Hz, 1H), 6.67 (d, J = 16.2 Hz, 1H), 6.49-6.41 (m, 2H), 6.15-6.02 (m, 1H), 3.82 (s, 3H), 3.80 (s, 3H), 2.56-2.51 (m, 4H).

^{13}C-NMR (75 MHz, $CDCl_3$) δ 179.7, 160.1, 157.5, 127.3, 126.5, 125.5, 119.4 104.8, 98.4, 55.4, 55.3, 34.2, 28.4.

ESI HRMS m/z $(M+Na)^+$ calculated 259.0941, observed 259.0941.

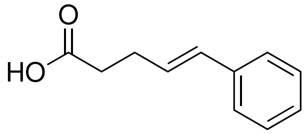

(4E)-5-phenylpent-4-enoic acid, 19a

To a solution of (3-carboxypropyl)triphenylphosphonium bromide (1.9 g, 4.5 mmol, 1.1 eq.) in tetrahydrofuran (20 ml) was added dropwise a solution of sodium bis(trimethylsilyl)amide (1.0 M in tetrahydrofuran, 9.0 ml, 9.0 mmol, 2.2 eq.) at 0 °C. The solution was stirred for 30 min, then cooled to -78 °C. Benzaldehyde (0.42 ml, 4.2 mmol, 1 eq.) was then added dropwise. The reaction was allowed to warm to room temperature overnight. Water and ether were added. The water layer was separated and acidified with 1 M aqueous hydrochloric acid to pH = 1, then extracted twice with ethyl acetate. The combined organic layers were dried over sodium sulfate, filtered and concentrated in vacuo. The crude product was chromatographed on silica gel (3:1 ether:hexanes) to give 0.70 g of product (95%) as a white solid.

IR (neat, cm^{-1}) 3024, 2356, 1694.

^{1}H-NMR (300 MHz, $CDCl_3$) δ 7.37-7.18 (m, 5H), 6.45 (d, J = 15.6 Hz, 1H), 6.27-6.17 (m, 1H), 2.56-2.53 (m, 4H).

^{13}C-NMR (75 MHz, $CDCl_3$) δ 179.8, 137.5, 131.5, 128.8, 128.2, 127.5, 126.4, 34.1, 28.1.

ESI HRMS m/z $(M+H)^+$ calculated 177.0910, observed 177.0910.

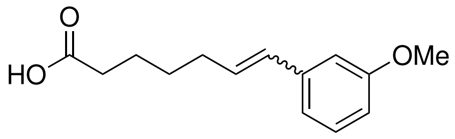

7-(3-methoxyphenyl)hept-6-enoic acid, 19h

To a solution of (5-carboxypentyl)triphenylphosphonium bromide (0.58 g, 1.3 mmol, 1.2 eq.) in tetrahydrofuran (6 ml) was added dropwise a solution of sodium bis(trimethylsilyl)amide (1.0 M in THF, 2.5 ml,

2.5 mmol, 2.3 eq.) at 0 °C. The solution was stirred for 30 min, then cooled to -78 °C. *M*-anisaldehyde (0.13 ml, 1.1 mmol, 1 eq.) was then added dropwise. The reaction was allowed to warm to room temperature overnight. Water and ether were added. The water layer was separated and acidified with 1 M hydrochloric acid to pH = 1, then extracted twice with ethyl acetate. The combined organic layers were dried over sodium sulfate, filtered and concentrated in vacuo. The crude product was chromatographed on silica gel (2:1 ether:hexanes) to give 0.23 g of product (92%) as a colorless oil.

IR (neat, cm^{-1}) 2938, 2673, 2360, 2343, 1705, 1598, 1578.

^{1}H-NMR (300 MHz, $CDCl_3$) δ 7.21 (dt, J(d) = 12.6, J(t) = 7.8 Hz, 1H), 6.96-6.71 (m, 3H), 6.39 (d, J = 11.4 Hz, 0.5H), 6.35 (d, J = 15.6 Hz, 0.5H), 6.18 (dt, J(d) = 15.6, J(t) = 6.9 Hz, 0.5H), 5.63 (dt, J(d) = 11.4 Hz, J(t) = 7.2 Hz, 0.5H), 3.78 (s, 3H), 2.43-2.29 (m, 3H), 2.22 (q, J = 6.6 Hz, 1H), 1.75-1.59 (m, 2H), 1.57-1.42 (m, 2H).

^{13}C-NMR (75 MHz, $CDCl_3$) δ 180.60, 180.56, 159.9, 159.5, 139.3, 139.1, 132.7, 130.7, 130.3, 129.6, 129.3, 129.2, 121.4, 118.8, 114.5, 112.7, 112.2, 111.4, 55.3, 34.1, 34.0, 32.7, 29.4, 28.8, 28.4, 24.4, 24.3.

ESI HRMS m/z $(M+H)^+$ calculated 235.1329, observed 235.1325.

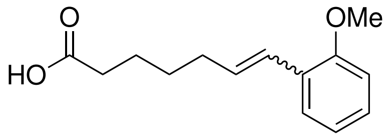

7-(2-methoxyphenyl)hept-6-enoic acid, 19f

To a solution of (5-carboxypentyl)triphenylphosphonium bromide (0.57 g, 1.2 mmol, 1.2 eq.) in tetrahydrofuran (6 ml) was added dropwise a solution of sodium bis(trimethylsilyl)amide (1.0 M in tetrahydrofuran, 2.5 ml, 2.5 mmol, 2.5 eq.) at 0 °C. The solution was stirred for 30 min, then cooled to -78 °C. A solution of *o*-anisaldehyde (0.14 g, 1.0 mmol, 1 eq.) in tetrahydrofuran (3 mL) was then added dropwise. The reaction was allowed to warm to rt overnight. Water and ether were added. The water layer was separated and acidified with 1 M hydrochloric acid to pH = 1, then extracted twice with ethyl acetate. The combined organic layers were dried over sodium sulfate, filtered and concentrated in vacuo. The crude product was chromatographed on silica gel (2:1 ether:hexanes) to give 0.20 g of product (82%) as a colorless oil.

IR (neat, cm^{-1}) 2936, 2673, 1707, 1597, 1578.

^{1}H-NMR (300 MHz, $CDCl_3$) δ .41 (dd, J = 7.5, 1.8 Hz, 0.4H), 7.27-7.14 (m, 1.6H), 6.96-6.82 (m, 2H), 6.71 (d, J = 15.9 Hz, 0.4H), 6.53 (d, J = 11.7 Hz, 0.6H), 6.19 (dt, Jd =15.9, J(t) = 6.9 Hz, 0.4H), 5.70 (dt, J(d) = 11.7, J(t) = 7.2 Hz, 0.6H), 3.84 (s, 1.2H), 3.83 (s, 1.8 H), 2.44-2.20 (m, 4H), 1.77-1.60 (m, 2H), 1.60-1.41 (m, 2H).

^{13}C-NMR (75 MHz, $CDCl_3$) δ 180.63, 180.59, 157.1, 156.4, 132.3, 131.1, 130.1, 128.3, 128.1, 126.9, 126.53, 126.45, 124.9, 124.8, 120.8, 120.2, 110.9, 110.5, 55.57, 55.54, 34.14, 34.07, 33.2, 29.4, 29.0, 28.4, 24.5, 24.4.

ESI HRMS m/z $(M+Na)^+$ calculated 257.1148, observed 257.1143.

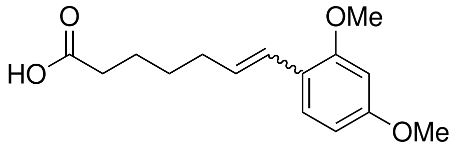

7-(2,4-dimethoxyphenyl)hept-6-enoic acid, 19j

To a solution of (5-carboxypentyl)triphenylphosphonium bromide (0.57 g, 1.2 mmol, 1.1 eq.) in tetrahydrofuran (6 mL) was added dropwise a solution of sodium bis(trimethylsilyl)amide (1.0 M in tetrahydrofuran, 2.5 ml, 2.5 mmol, 2.4 eq.) at 0 °C. The solution was stirred for 30 min, then cooled to -78 °C. A solution of 2,4-dimethoxybenzaldehyde (0.175 g, 1.05 mmol, 1 eq.) in tetrahydrofuran (2 mL) was then added dropwise. The reaction was allowed to warm to room temperature overnight. Water and ether were added. The water layer was separated and acidified with 1 M hydrochloric acid to pH = 1, then extracted twice with ethyl acetate. The combined organic layers were dried over sodium sulfate, filtered and concentrated in vacuo. The crude product was chromatographed on silica gel (2:1 ether:hexanes) to give 0.220 g of product (79%)

as an oily solid.

IR (neat, cm^{-1}) 2937, 2666, 2343, 2362, 1707, 1608, 1578.

^{1}H-NMR (300 MHz, $CDCl_3$) 7.31 (d, J = 8.1 Hz, 0.6H), 7.13 (d, J = 9.0 Hz, 0.4H), 6.61 (d, J = 15.9 Hz, 0.6H), 6.51-6.40 (m, 2.4H), 6.07 (dt, J(d) = 15.9, J(t) = 6.9 Hz, 0.6H), 5.62 (dt, J(d) = 11.4, J(t) = 7.2 Hz, 0.4H), 3.82 (s, 3H), 3.80 (s, 3H), 2.36 (tt, J = 7.8 Hz, 2H), 2.30-2.18 (m, 2H), 1.78-1.60 (m, 2H), 1.58-1.41 (m, 2H).

^{13}C-NMR (75 MHz, $CDCl_3$) δ 180.58, 180.53, 160.0, 158.2, 157.4, 131.1, 130.4, 129.0, 127.2, 124.6, 124.3, 120.0, 119.3, 104.8, 103.8, 98.53, 98.47, 55.6, 55.5, 34.14, 34.07, 33.2, 29.4, 29.1, 28.4, 24.5, 24.4.

ESI HRMS m/z $(M+H)^+$ calculated 287.1254, observed 287.1251.

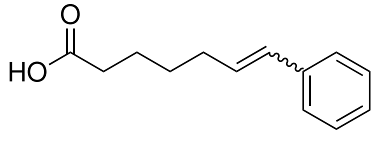

7-phenyl-6-heptenoic acid, 19b

To a suspension of (5-carboxypentyl)triphenylphosphonium bromide (4.228 g, 9.245 mmol, 1 eq.) in tetrahydrofuran (20 mL) was added dropwise a solution of sodium bis(trimethylsilyl)amide (1.0 M in tetrahydrofuran, 18.5 mL, 18.5 mmol, 2 eq.) at 0 °C. The solution was stirred for 30 min at 0 °C. A solution of benzaldehyde (0.94 mL, 9.245 mmol, 1 eq.) in tetrahydrofuran (3 mL) was then added dropwise. The reaction was allowed to warm to room temperature overnight. Water and ether were added. The water layer was separated and acidified with 3 M hydrochloric acid to pH = 1, then extracted three times with ethyl ether. The combined organic layers were dried over magnesium sulfate, filtered and concentrated in vacuo. The crude product was chromatographed on silica gel with a mixture of hexanes and ethyl acetate to give 1.242 g of product (66%) as a colorless oil and a 2:1 mixture of isomers.

The spectral data catalogued below matches that which has been previously reported for this compound (Henry-Riyad, H.; Tidwell, T. T. *Can. J. Chem.* **2003**, *81*, 697-704).

IR (neat, cm^{-1}) 3081, 3056, 3024, 2933, 2862, 2673, 1707, 1493, 1447, 1412.

^{1}H-NMR (300 MHz, $CDCl_3$) δ 7.41-7.14 (m, 5H), 6.43 (d, J = 10.5 Hz, 0.33H), 6.39 (d, J = 15.6 Hz, 0.67H, A of AB pattern), 6.19 (dt, J(d) = 15.6 Hz, J(t) = 6.6 Hz, 0.67H, B of AB pattern), 5.64 (dt, J(d) = 11.7 Hz, J(t) = 7.2 Hz), 2.44-2.30 (m, 2.67H), 2.23 (q, J = 7.5, 1.33H), 1.77-1.61 (m, 2H), 1.61-1.45 (m, 2H).

^{13}C-NMR (75 MHz, $CDCl_3$) δ 180.5, 137.7, 137.6, 132.2, 130.3, 130.2, 129.3, 128.7, 128.5, 128.1, 126.9, 126.5, 125.9, 34.0, 33.9, 32.6, 29.2, 28.7, 28.2, 24.24, 24.17.

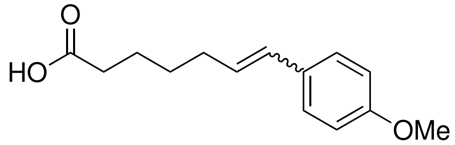

7-(4-methoxyphenyl)-6-Heptenoic acid, 19d

To a suspension of (5-carboxypentyl)triphenylphosphonium bromide (3.968 g, 8.676 mmol, 1 eq.) in tetrahydrofuran (20 mL) was added dropwise a solution of sodium bis(trimethylsilyl)amide (1.0 M in tetrahydrofuran, 17.4 mL, 17.4 mmol, 2 eq.) at 0 °C. The solution was stirred for 30 min at 0 °C. A solution of p-anisaldehyde (1.05 mL, 8.676 mmol, 1 eq.) in tetrahydrofuran (3 mL) was then added dropwise. The reaction was allowed to warm to room temperature overnight. Water and ether were added. The aqueous layer was washed three times with ether. The water layer was then acidified with 3 M hydrochloric acid to pH = 1 and extracted three times with ethyl ether. The combined organic layers were dried over magnesium sulfate, filtered and concentrated in vacuo. The crude product was chromatographed on silica gel with a mixture of hexanes and ethyl acetate to give 1.317 g of product (65%) as a light brown solid and a 2:1 mixture of isomers.

IR (neat, cm^{-1}) 3006, 2933, 2865, 2838, 2679, 1703, 1607, 1574, 1511, 1462, 1442, 1433, 1424, 1403.

^{1}H-NMR (300 MHz, CD_3OD) δ 7.26 (d, J = 8.7 Hz, 1.33H), 7.19 (d, J = 8.7 Hz, 0.67H), 6.7 (d, J = 8.7 Hz, 0.67H), 6.82 (d, J = 8.7 Hz, 1.33H), 6.34 (d, J = 11.4 Hz, 0.33H), 6.32 (d, J = 15.9 Hz, 0.66H, A of AB pattern), 6.06 (dt, J(d) = 15.6 Hz, J(t) = 6.9 Hz, 0.66H, B of AB pattern), 5.54 (dt, J(d) = 11.7, J(t) = 7.2, 0.33H), 3.77 (s, 1H), 3.76 (s, 2H), 2.37-2.15 (m, 4H), 1.71-1.57 (m, 2H), 1.56-1.42 (m, 2H).

^{13}C-NMR (75 MHz, $CDCl_3$) δ 180.4, 158.6, 158.2, 130.7, 130.5, 130.3, 129.9, 129.6, 128.7, 128.0, 127.0, 113.9, 113.6, 55.2, 34.0, 32.6, 29.3, 28.8, 28.2, 24.3, 24.2.

ESI HRMS m/z $(M+H)^+$ calculated 235.1329, observed 235.1329.

5 NMR Spectra

All ^{1}H-NMR and ^{13}C-NMR spectra for the compounds reported in this publication are presented in the following pages. Unless otherwise noted, all spectra were collected using a deuterated chloroform solvent.

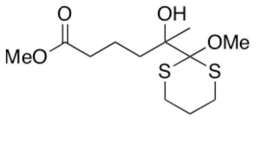
OH
OMe
MeO
O
S
S

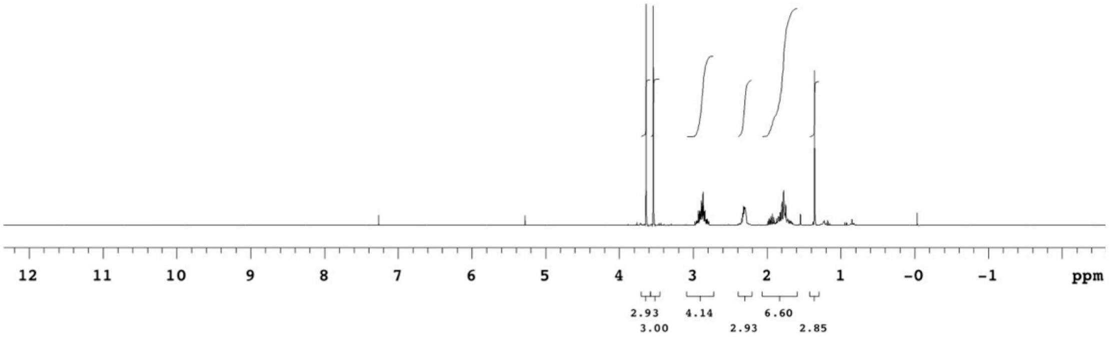
12 11 10 9 8 7 6 5 4 3 2 1 -0 -1 ppm
2.93
3.00
4.14
2.93
6.60
2.85

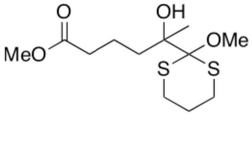
OH
OMe
MeO
O
S
S

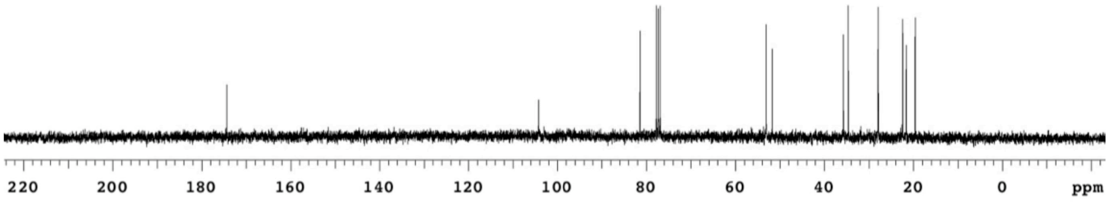
220 200 180 160 140 120 100 80 60 40 20 0 ppm

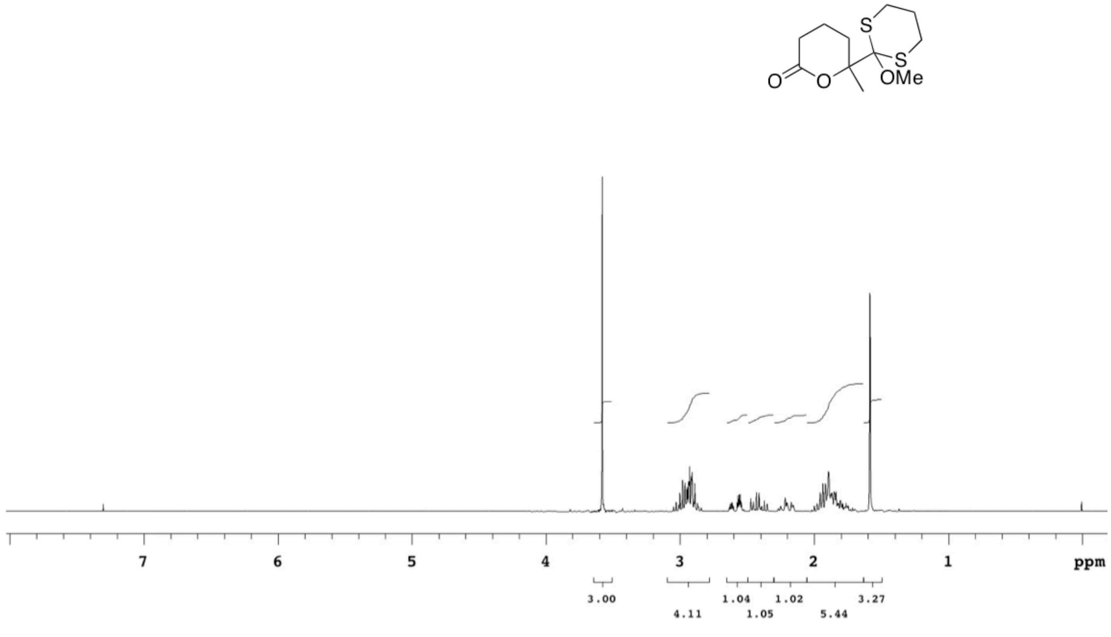
S
S
OMe
O
O
7
6
5
4
3
2
1
ppm
3.00
4.11
1.04
1.05
1.02
5.44
3.27

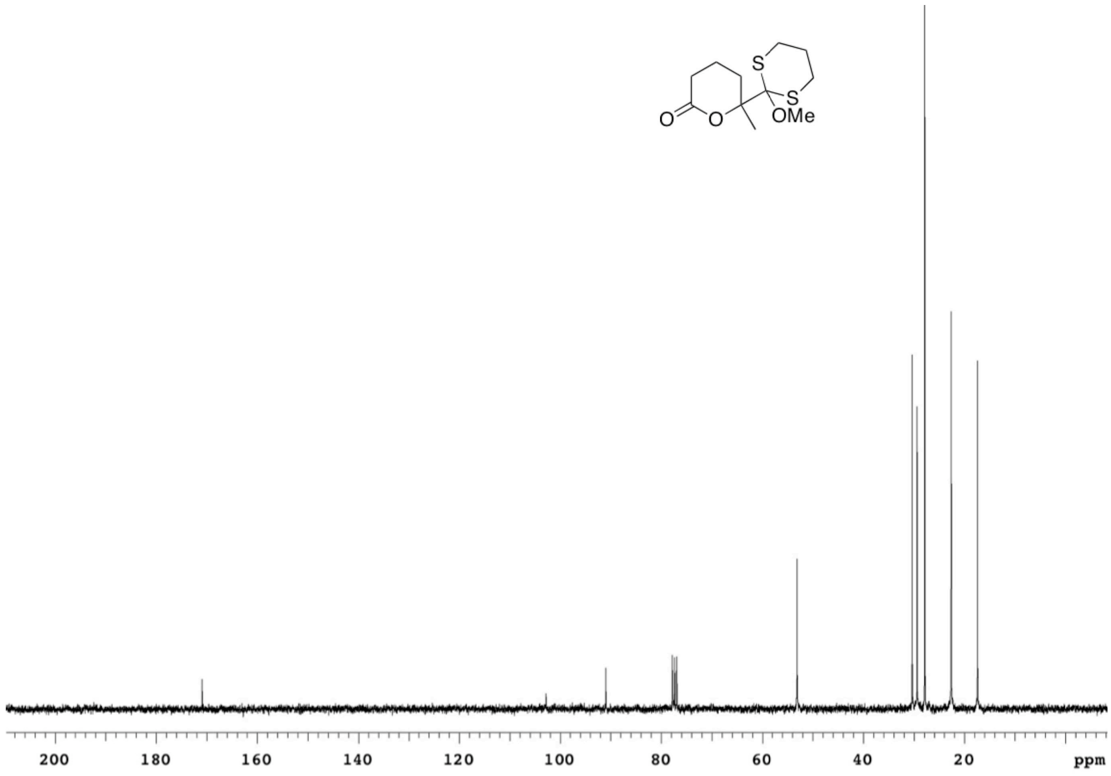
S
S
OMe
O
O
200
180
160
140
120
100
80
60
40
20
ppm

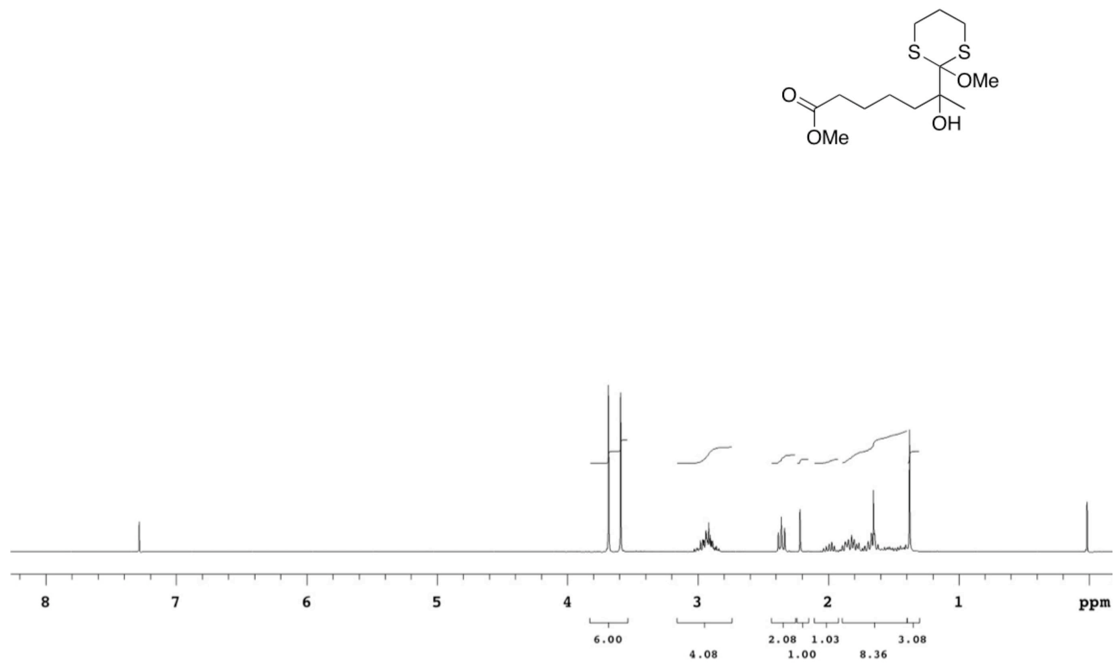
S
S
OMe
O
OMe
OH
8
7
6
5
4
3
2
1
ppm
6.00
4.08
2.08
1.00
1.03
8.36
3.08

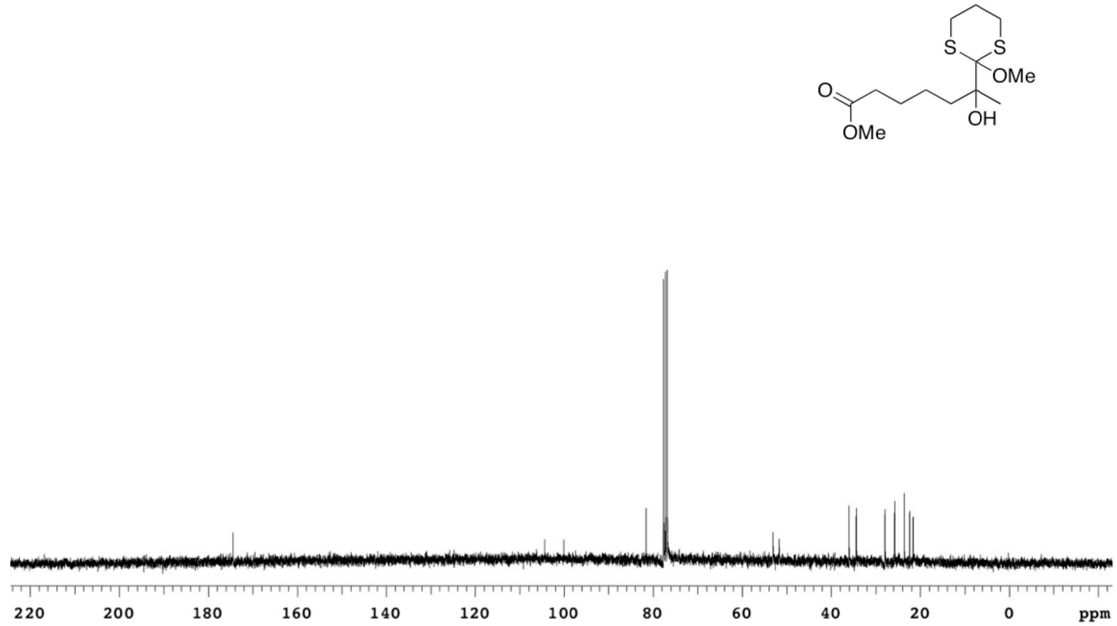
S
S
OMe
O
OMe
OH
220
200
180
160
140
120
100
80
60
40
20
0
ppm

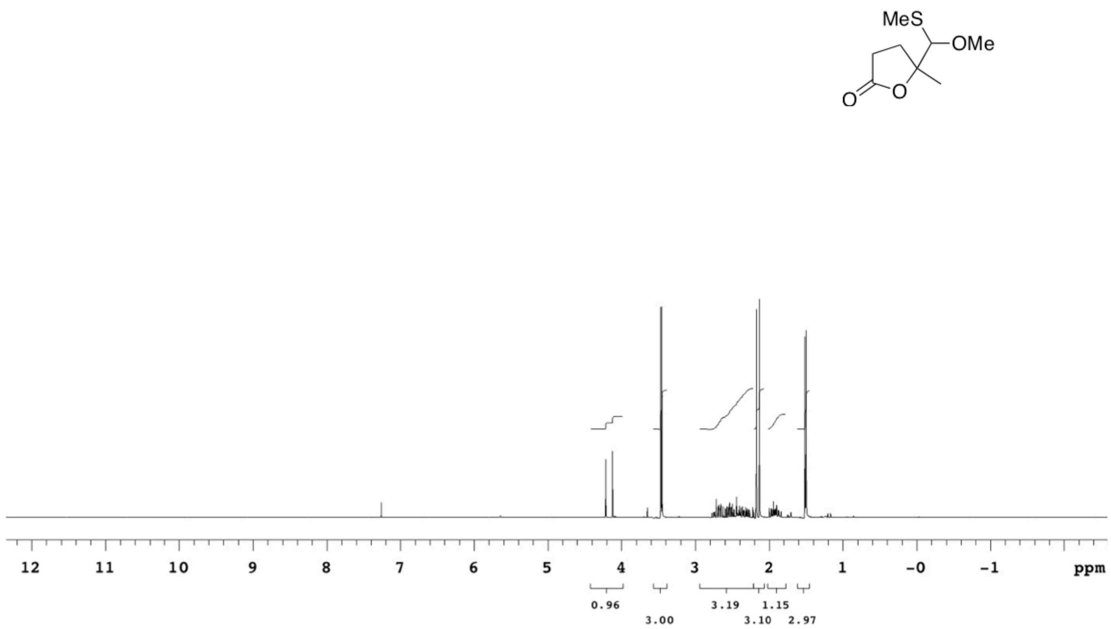
MeS
OMe
O
O
ppm
0.96
3.00
3.19
3.10
1.15
2.97

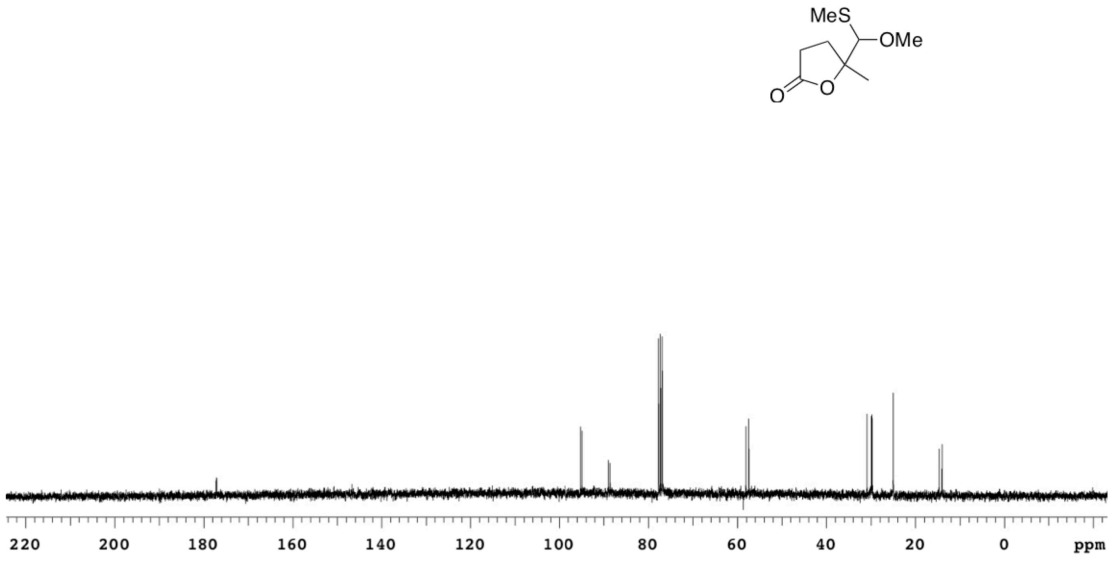
MeS
OMe
O
O
ppm

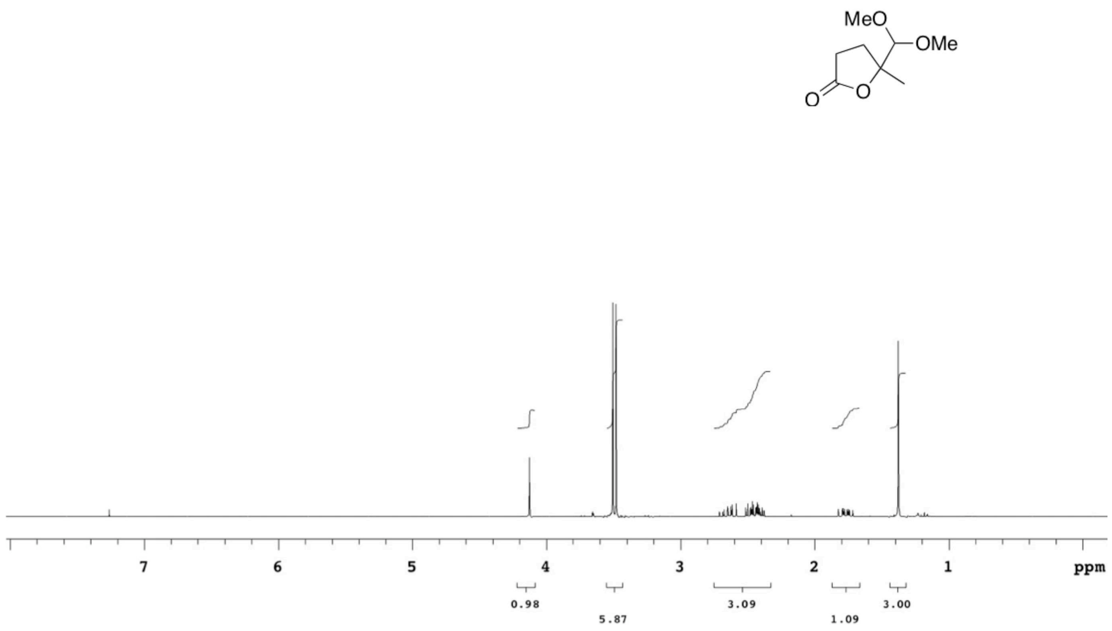
MeO
OMe
O
O
7 6 5 4 3 2 1 ppm
0.98 5.87 3.09 1.09 3.00

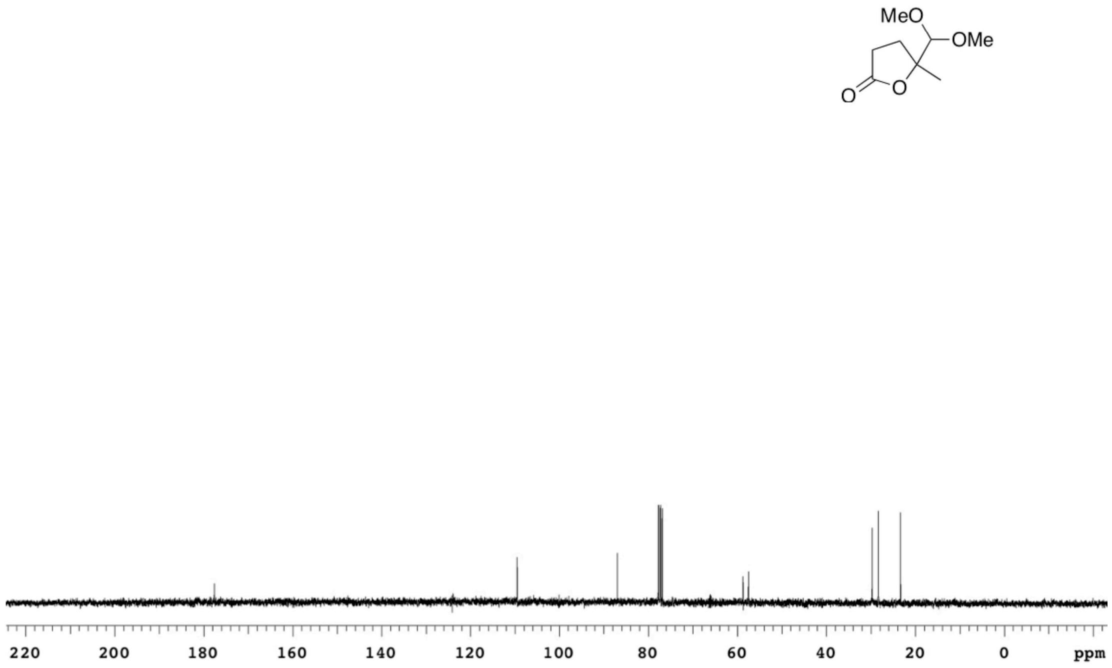
MeO
OMe
O
O
220 200 180 160 140 120 100 80 60 40 20 0 ppm

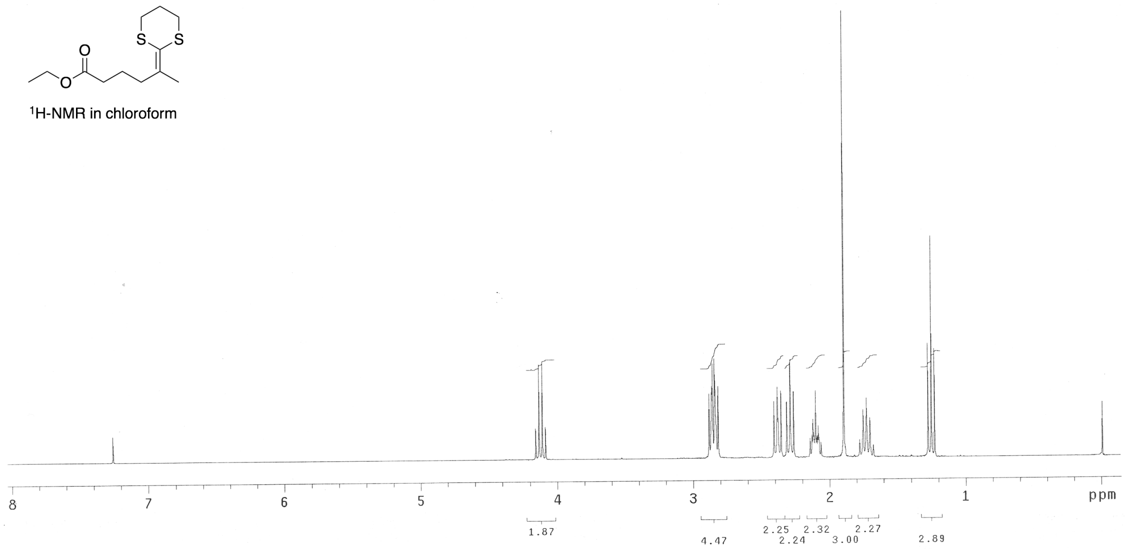
1H-NMR in chloroform
8 7 6 5 4 3 2 1 ppm
1.87
4.47
2.25 2.24 2.32 3.00 2.27
2.89

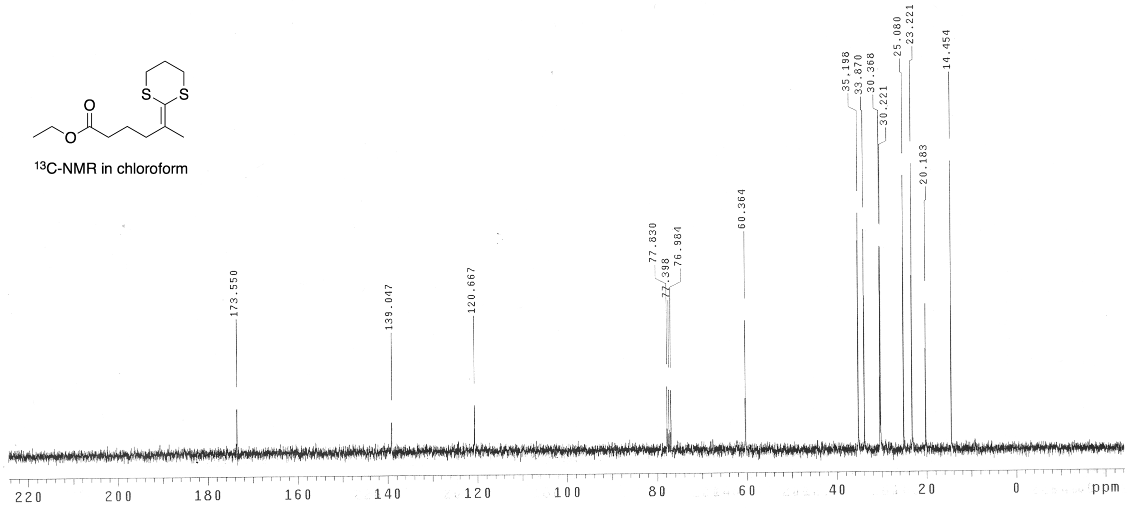
13C-NMR in chloroform
173.550
139.047
120.667
77.830
77.398
76.984
60.364
35.198
33.870
30.368
30.221
25.080
23.221
20.183
14.454
220 200 180 160 140 120 100 80 60 40 20 0 ppm

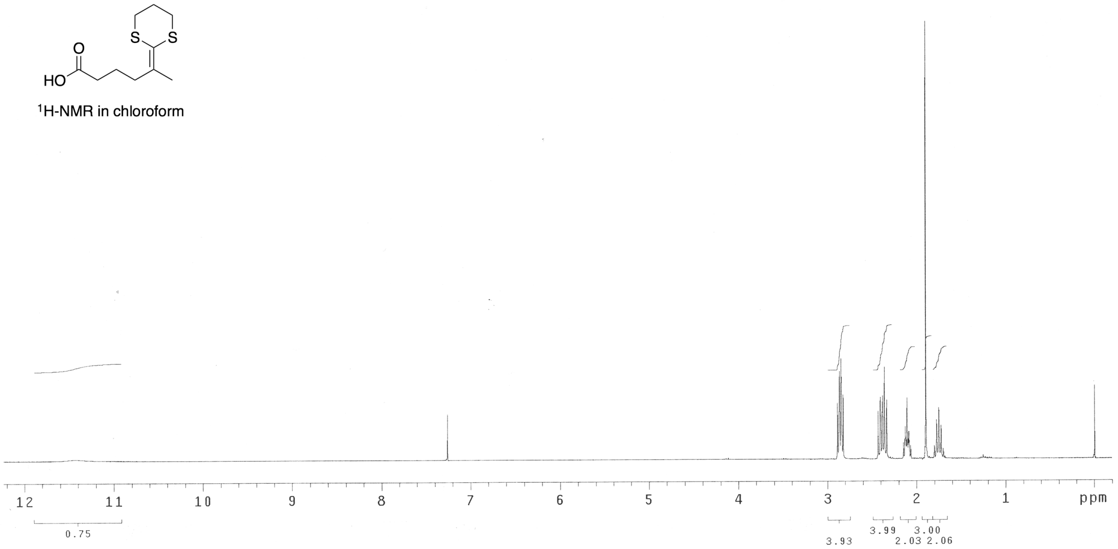

HO
O
S
S
1H-NMR in chloroform
12
11
10
9
8
7
6
5
4
3
2
1
ppm
0.75
3.93
3.99
2.03
3.00
2.06

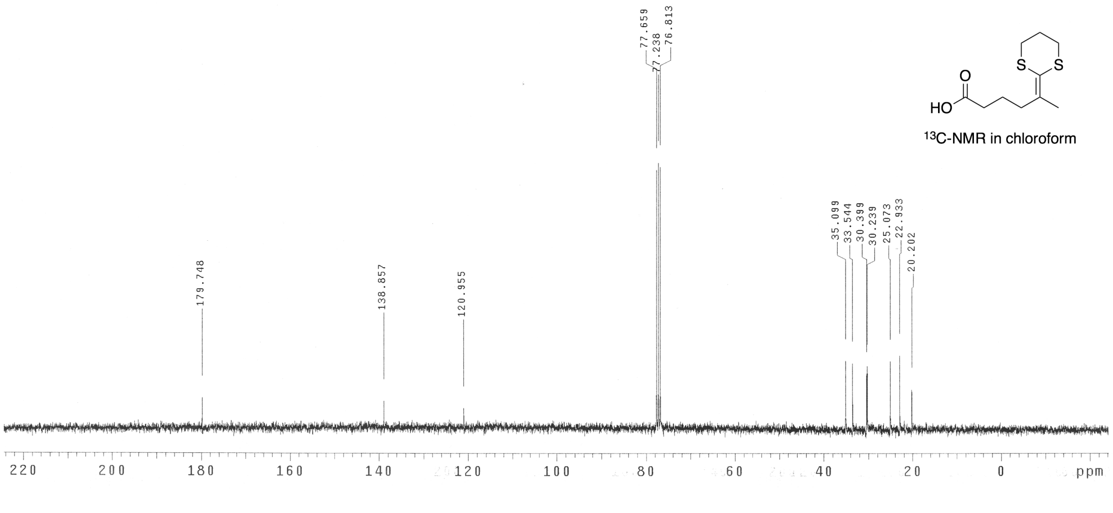

HO
O
S
S
13C-NMR in chloroform
77.659
77.238
76.813
179.748
138.857
120.955
35.099
33.544
30.399
30.239
25.073
22.933
20.202
220
200
180
160
140
120
100
80
60
40
20
0
ppm

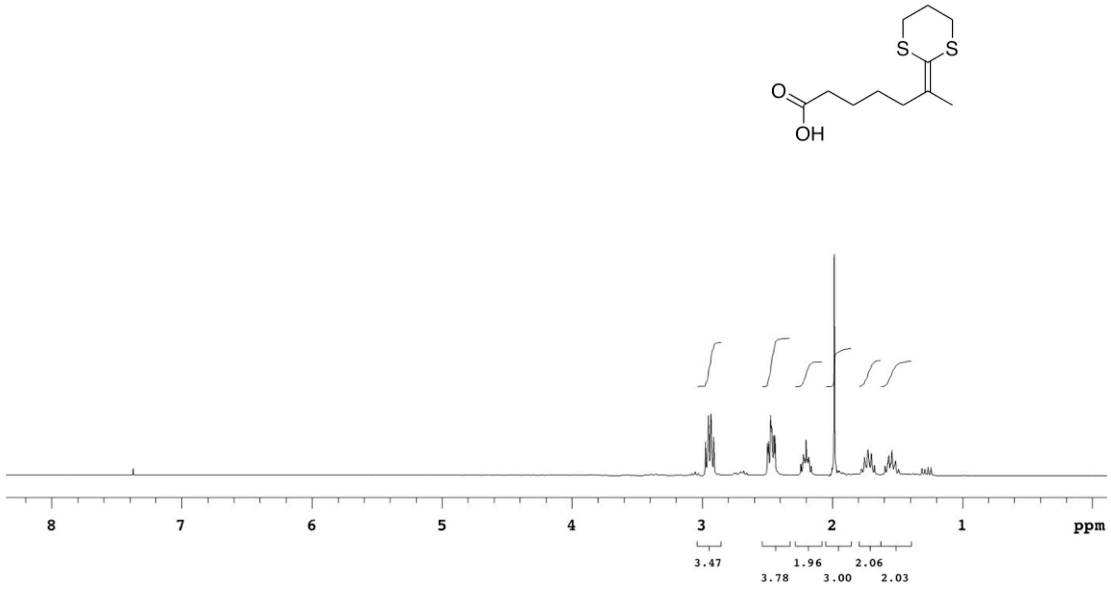
S
S
O
OH
8
7
6
5
4
3
2
1
ppm
3.47
3.78
1.96
3.00
2.06
2.03

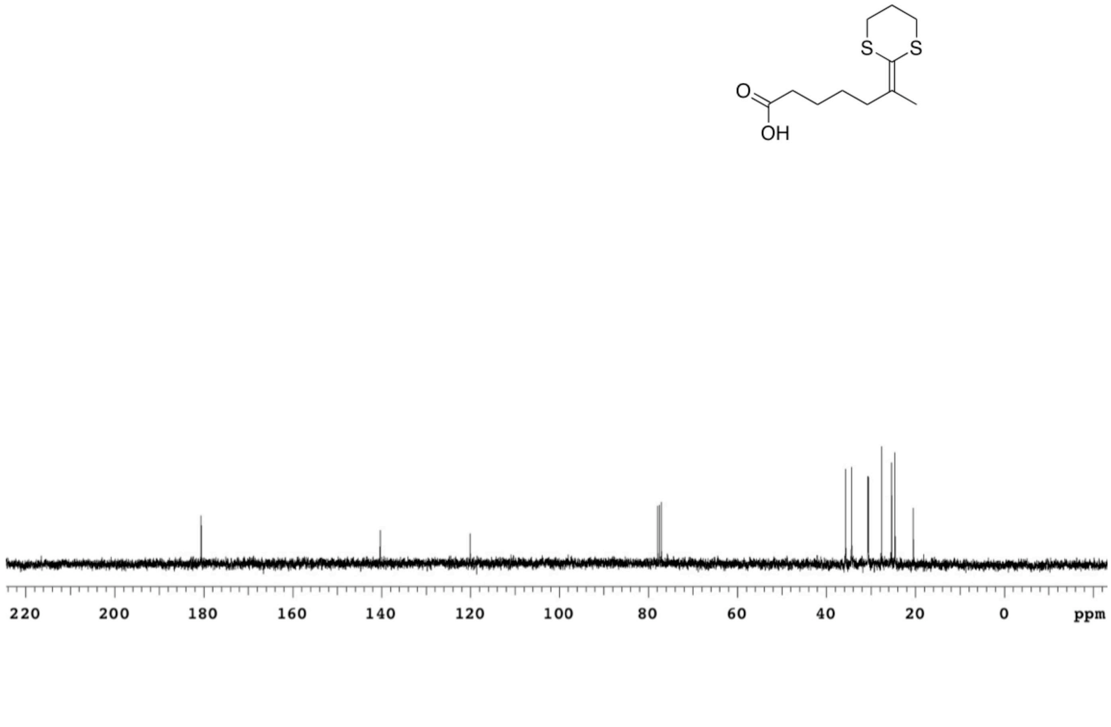
S
S
O
OH
220
200
180
160
140
120
100
80
60
40
20
0
ppm

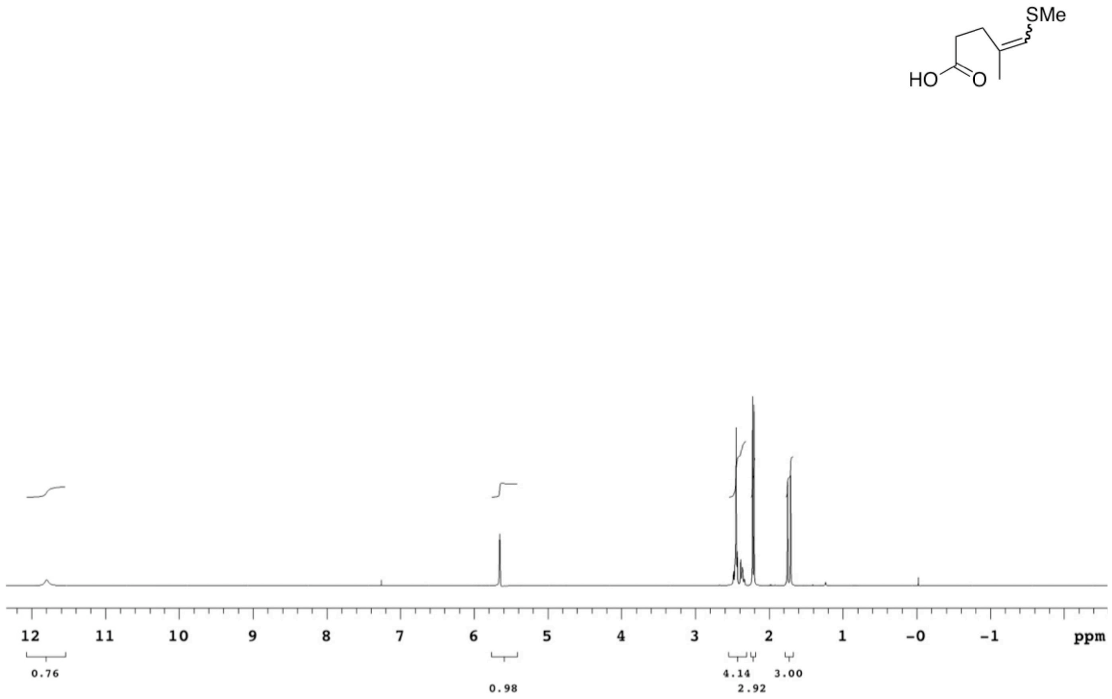
SMe
HO
O
12 11 10 9 8 7 6 5 4 3 2 1 −0 −1 ppm
0.76
0.98
4.14
2.92
3.00

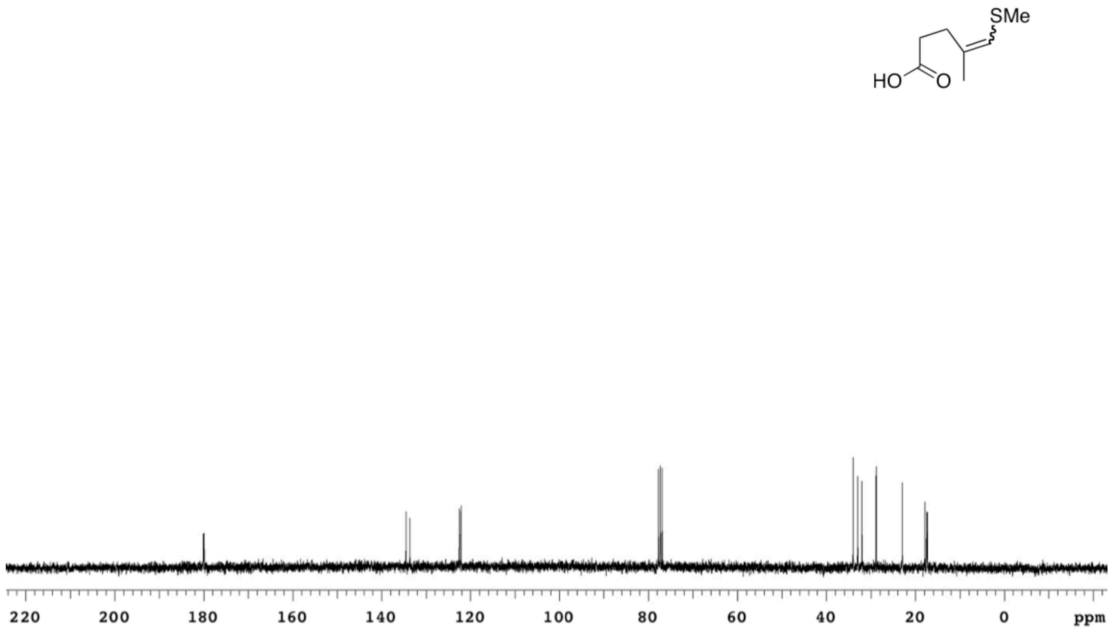
SMe
HO
O
220 200 180 160 140 120 100 80 60 40 20 0 ppm

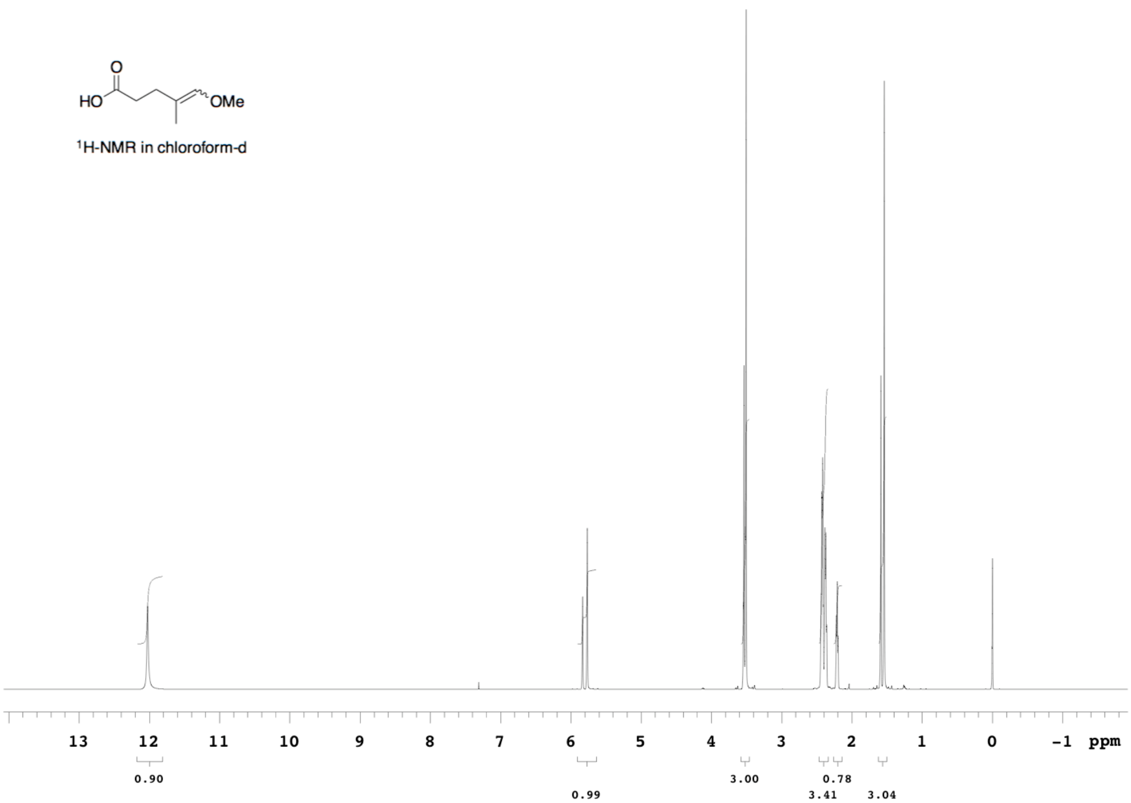

HO
O
OMe
¹H-NMR in chloroform-d
13 12 11 10 9 8 7 6 5 4 3 2 1 0 -1 ppm
0.90
0.99
3.00
0.78
3.41
3.04

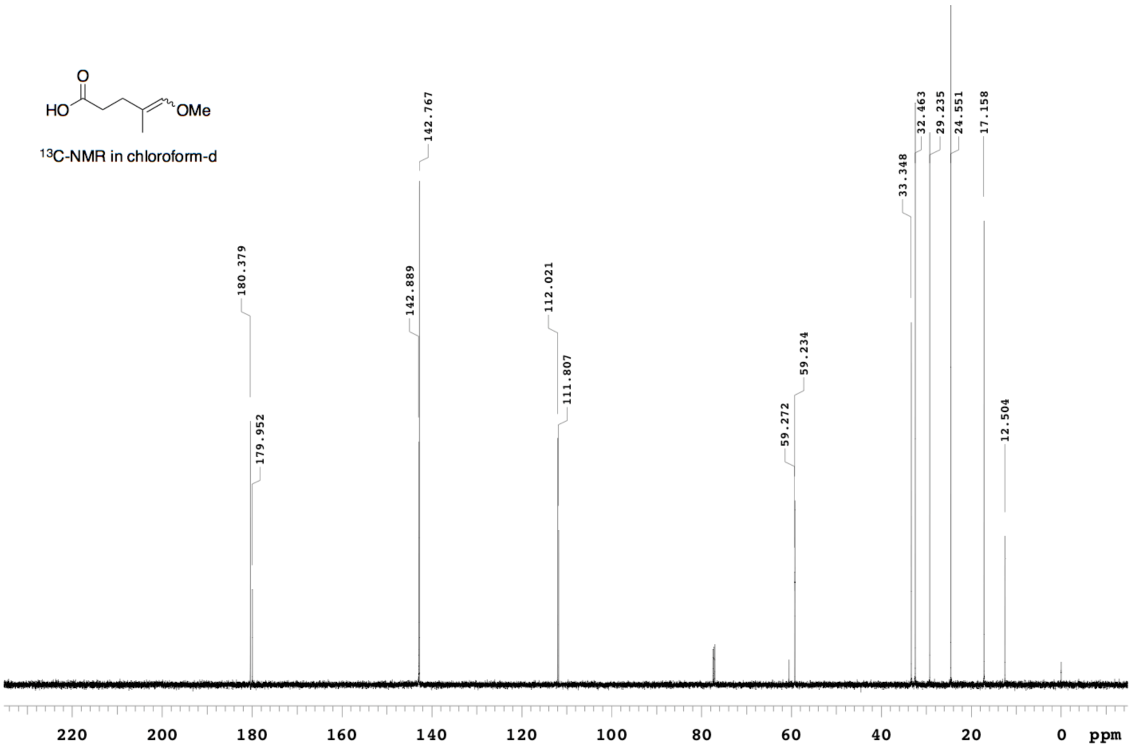

HO
O
OMe
¹³C-NMR in chloroform-d
180.379
179.952
142.889
142.767
112.021
111.807
59.272
59.234
33.348
32.463
29.235
24.551
17.158
12.504
220 200 180 160 140 120 100 80 60 40 20 0 ppm

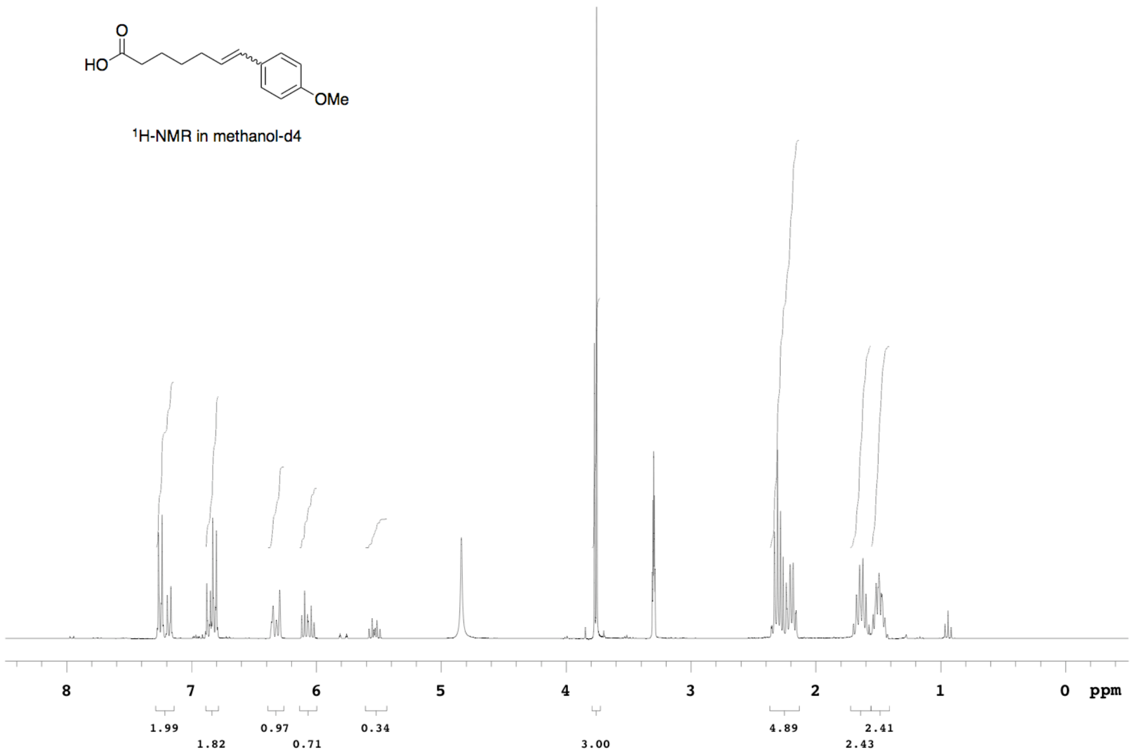
HO
O
OMe
1H-NMR in methanol-d4
8
7
6
5
4
3
2
1
0
ppm
1.99
1.82
0.97
0.71
0.34
3.00
4.89
2.43
2.41

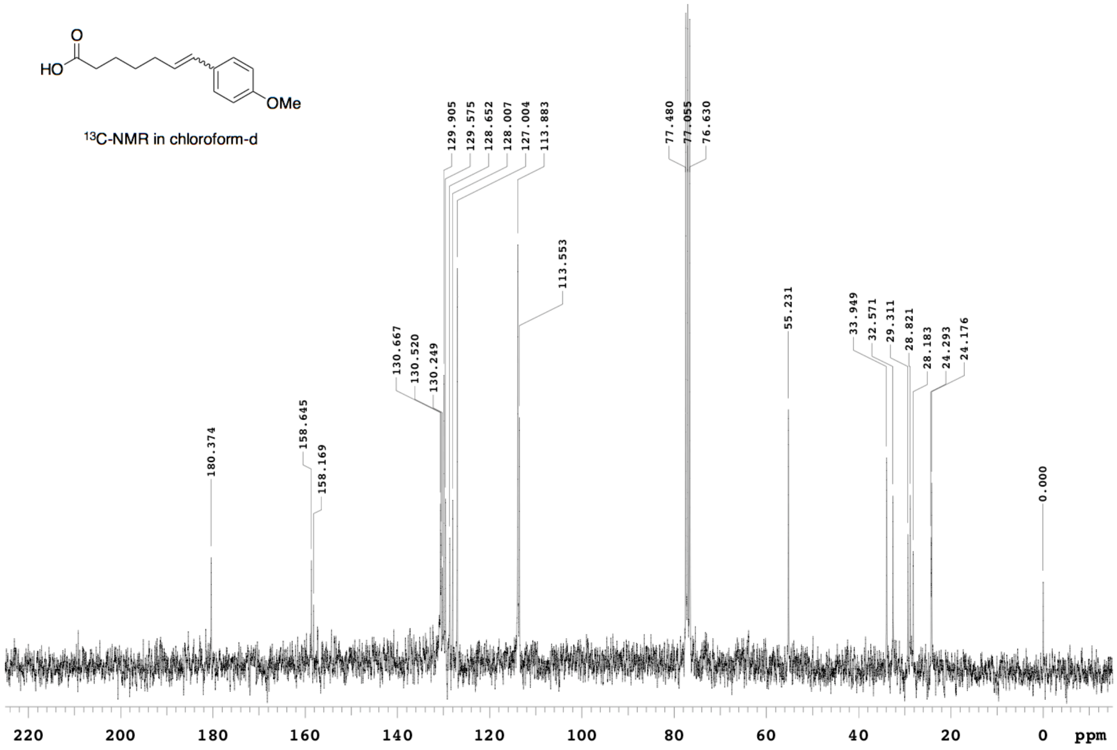
HO
O
OMe
13C-NMR in chloroform-d
180.374
158.645
158.169
130.667
130.520
130.249
129.905
129.575
128.652
128.007
127.004
113.883
113.553
77.480
77.055
76.630
55.231
33.949
32.571
29.311
28.821
28.183
24.293
24.176
0.000
220
200
180
160
140
120
100
80
60
40
20
0
ppm

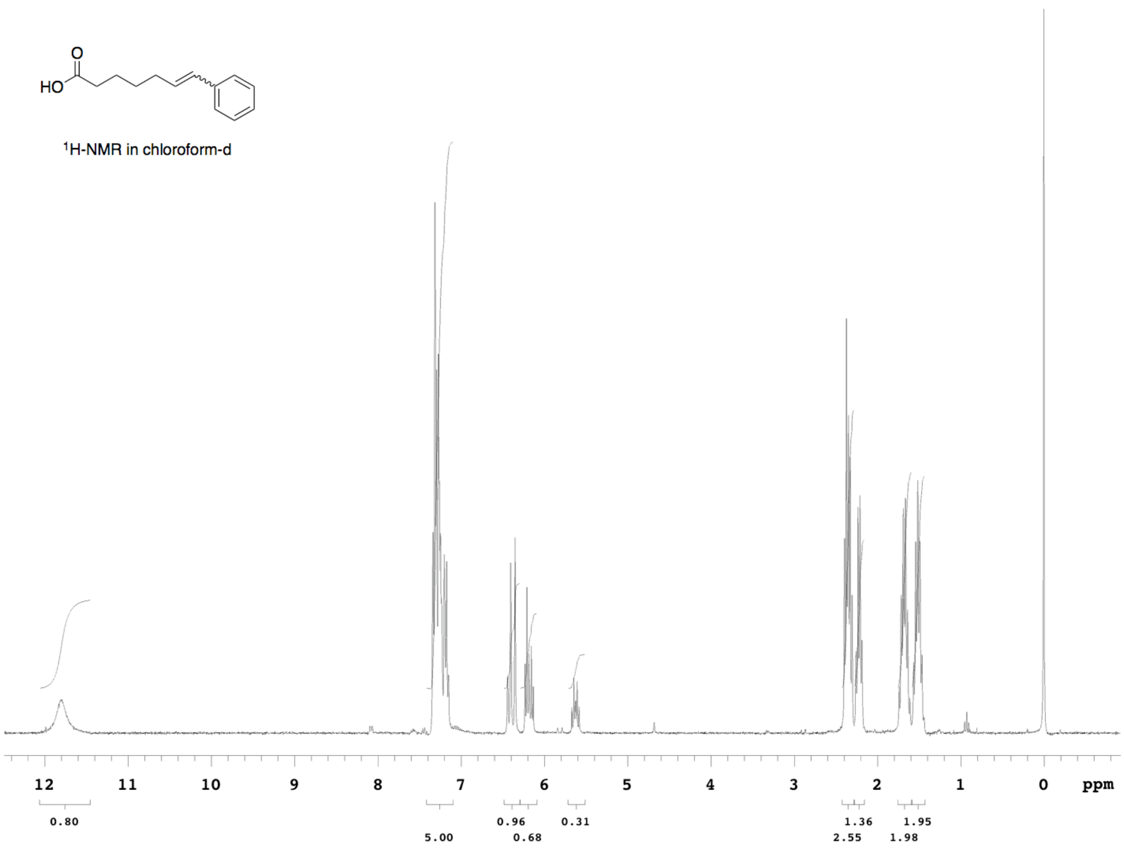
HO
O
1H-NMR in chloroform-d
12 11 10 9 8 7 6 5 4 3 2 1 0 ppm
0.80
5.00
0.96
0.68
0.31
1.36
2.55
1.95
1.98

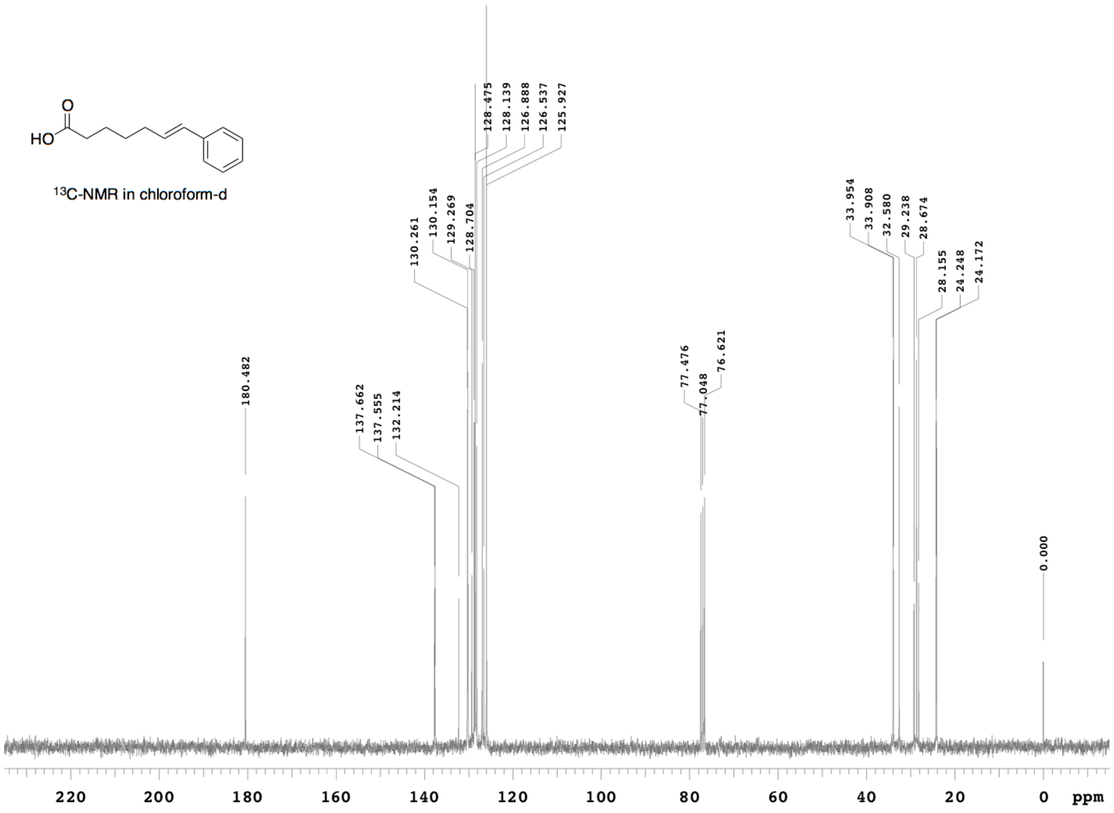
HO
O
13C-NMR in chloroform-d
180.482
137.662
137.555
132.214
130.261
130.154
129.269
128.704
128.475
128.139
126.888
126.537
125.927
77.476
77.048
76.621
33.954
33.908
32.580
29.238
28.674
28.155
24.248
24.172
0.000
220 200 180 160 140 120 100 80 60 40 20 0 ppm

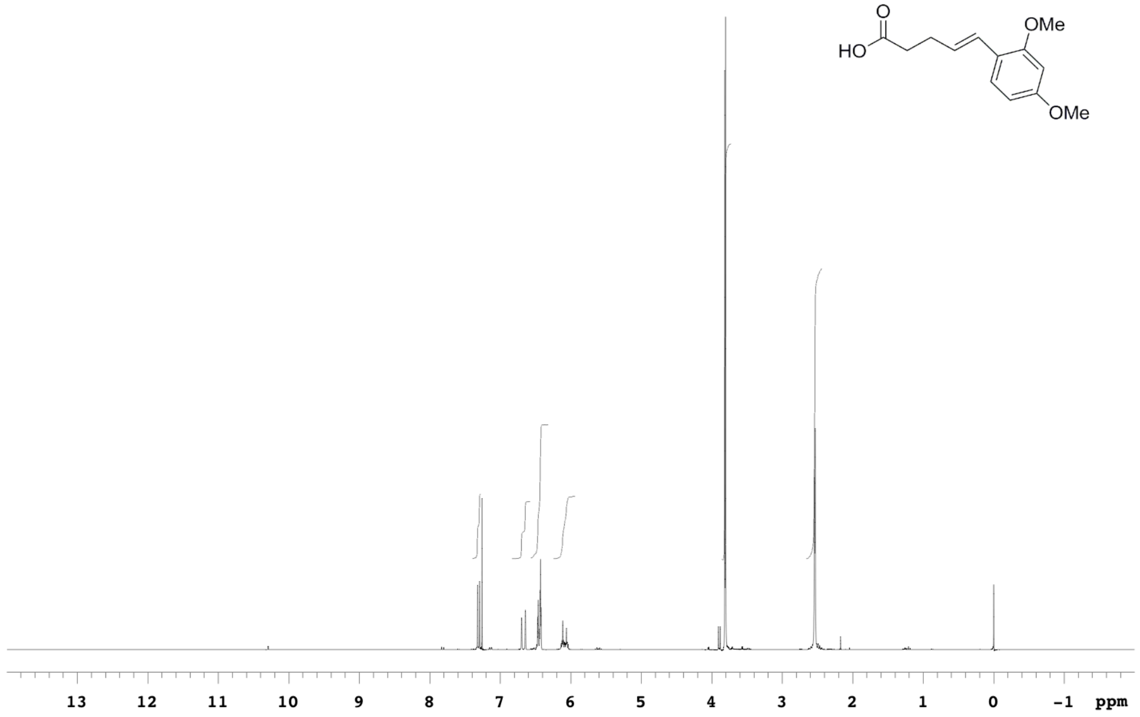
O
HO
OMe
OMe
13 12 11 10 9 8 7 6 5 4 3 2 1 0 -1 ppm

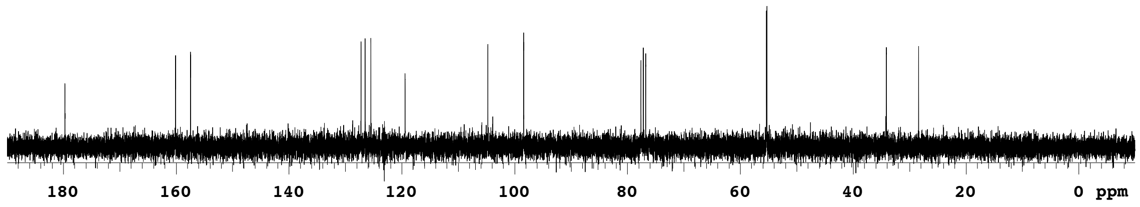
180 160 140 120 100 80 60 40 20 0 ppm

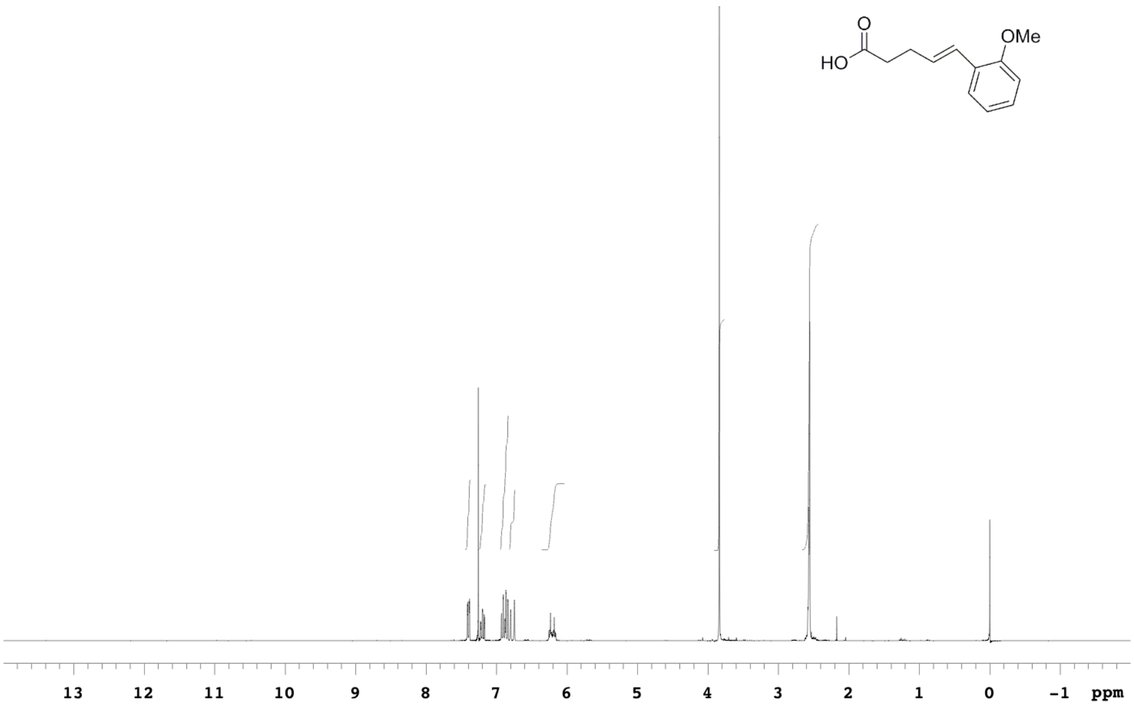

O
HO
OMe
13
12
11
10
9
8
7
6
5
4
3
2
1
0
-1
ppm

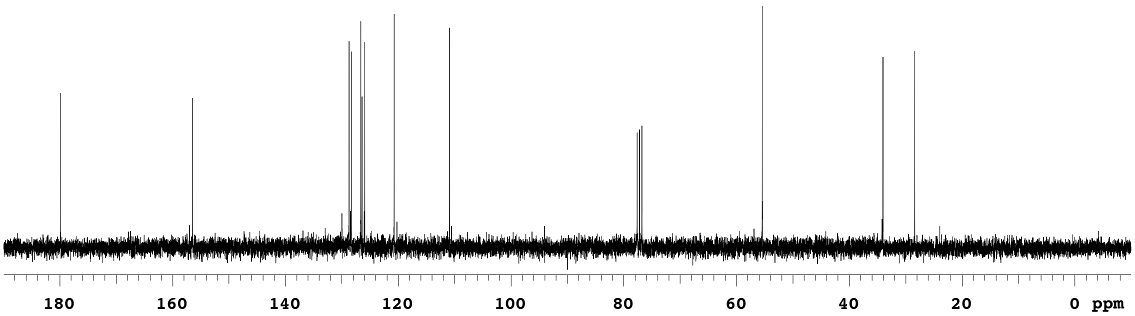

180
160
140
120
100
80
60
40
20
0
ppm

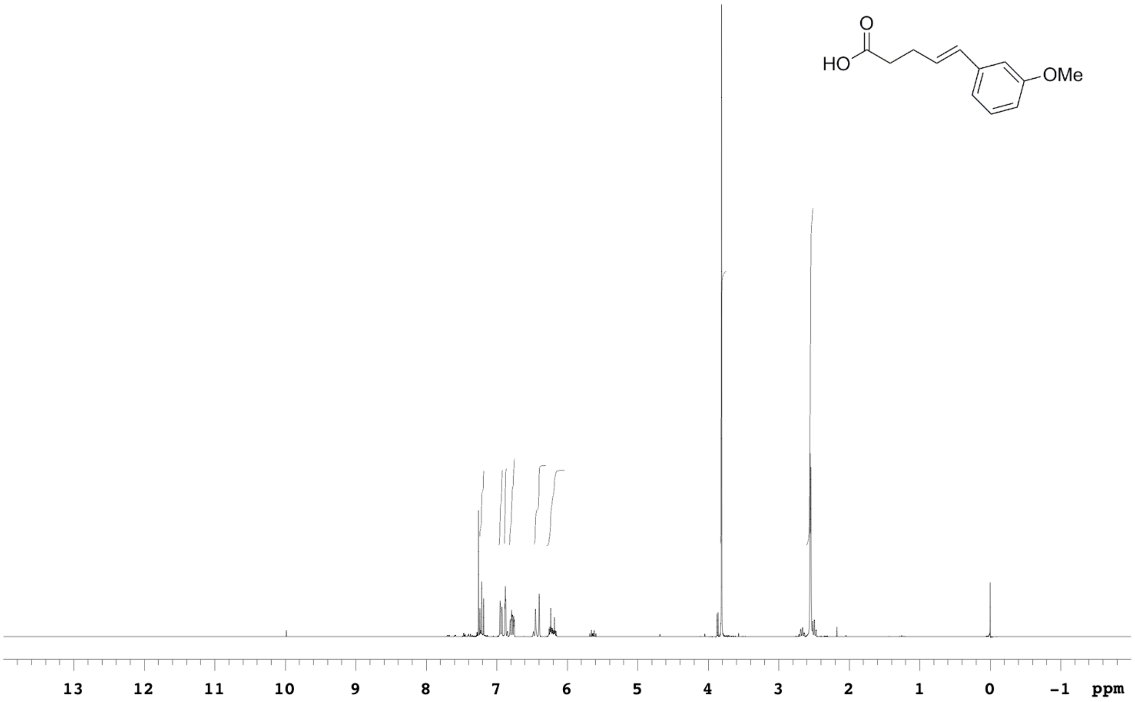
O
HO
OMe
13 12 11 10 9 8 7 6 5 4 3 2 1 0 -1 ppm

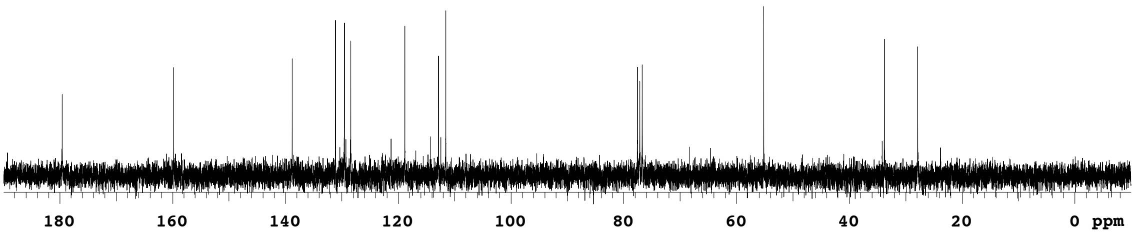
180 160 140 120 100 80 60 40 20 0 ppm

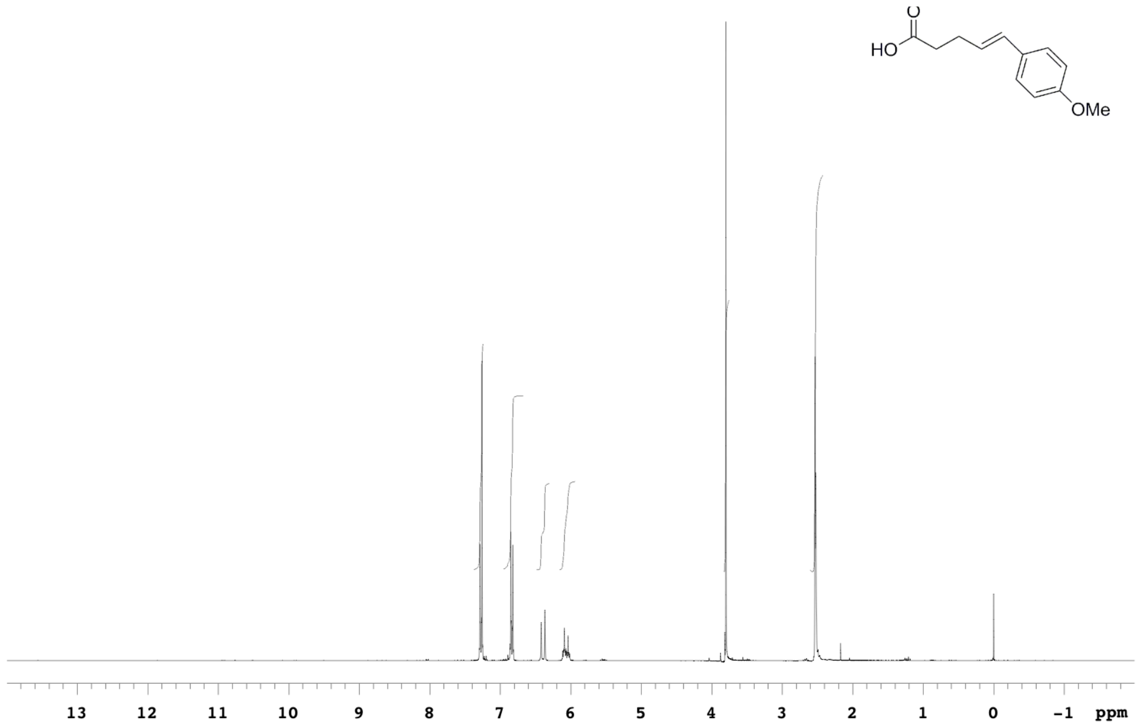
HO
O
OMe
13 12 11 10 9 8 7 6 5 4 3 2 1 0 -1 ppm

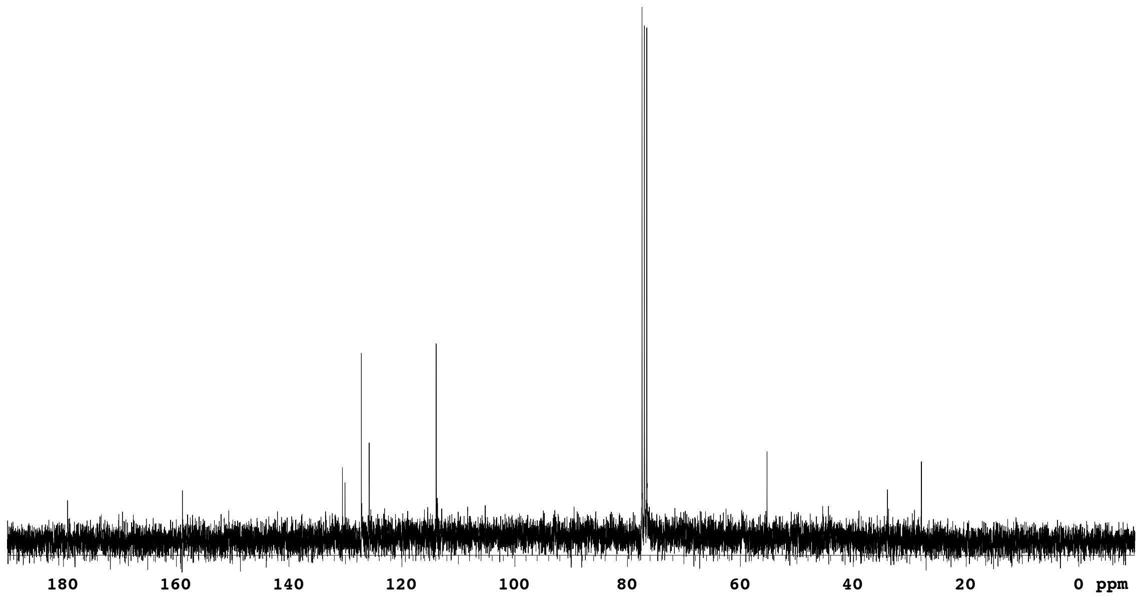
180 160 140 120 100 80 60 40 20 0 ppm

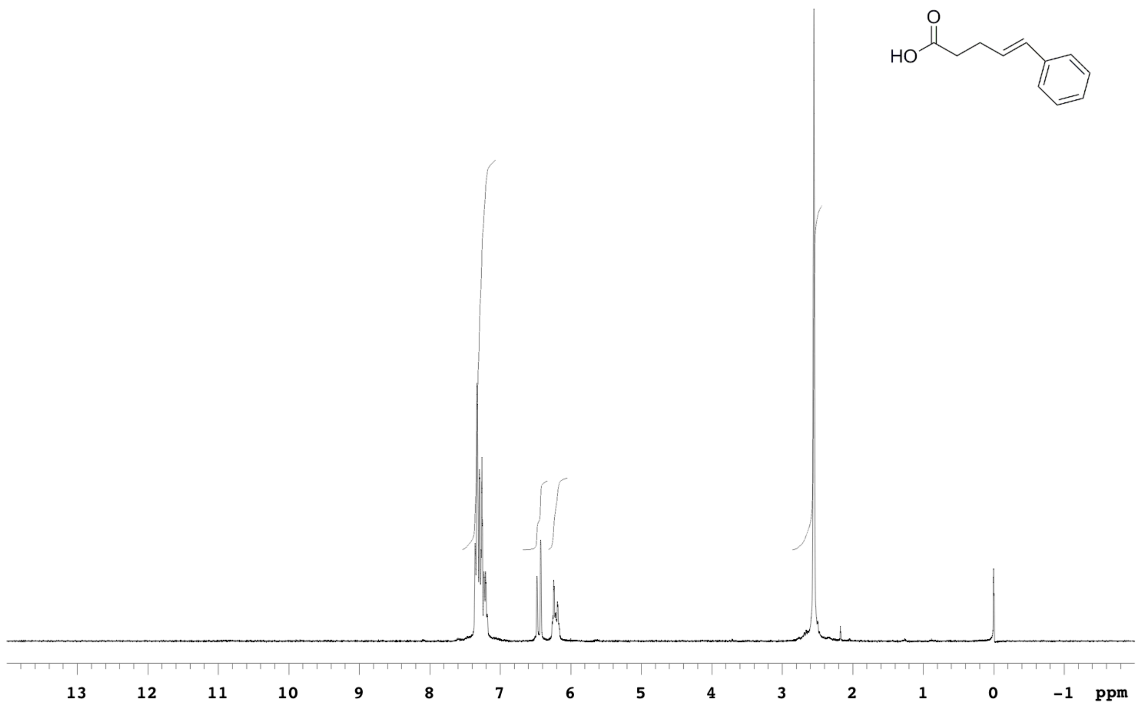

HO
O
13
12
11
10
9
8
7
6
5
4
3
2
1
0
-1
ppm

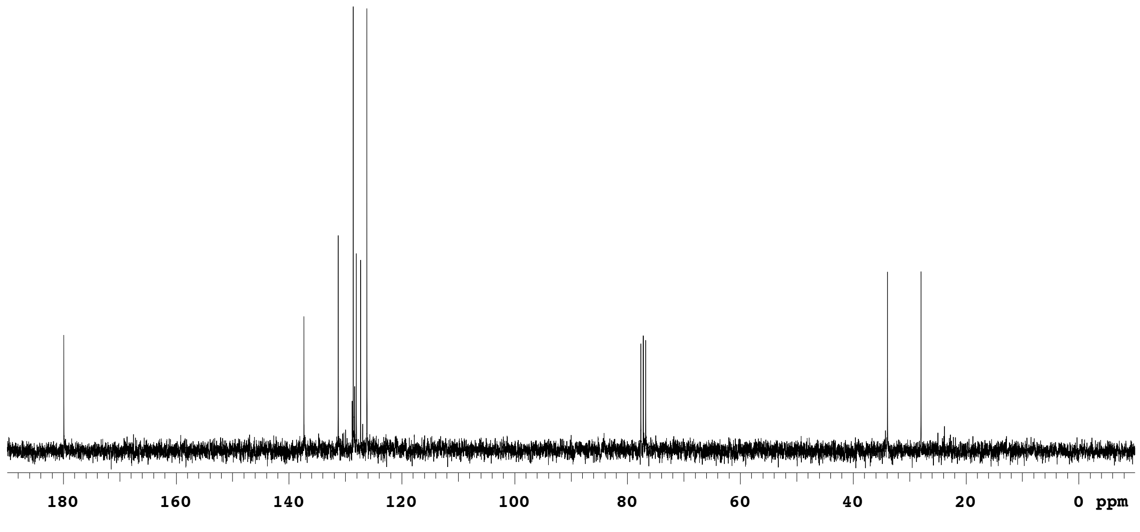

180
160
140
120
100
80
60
40
20
0
ppm

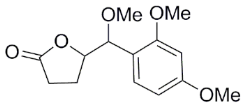

Major Diastereomer

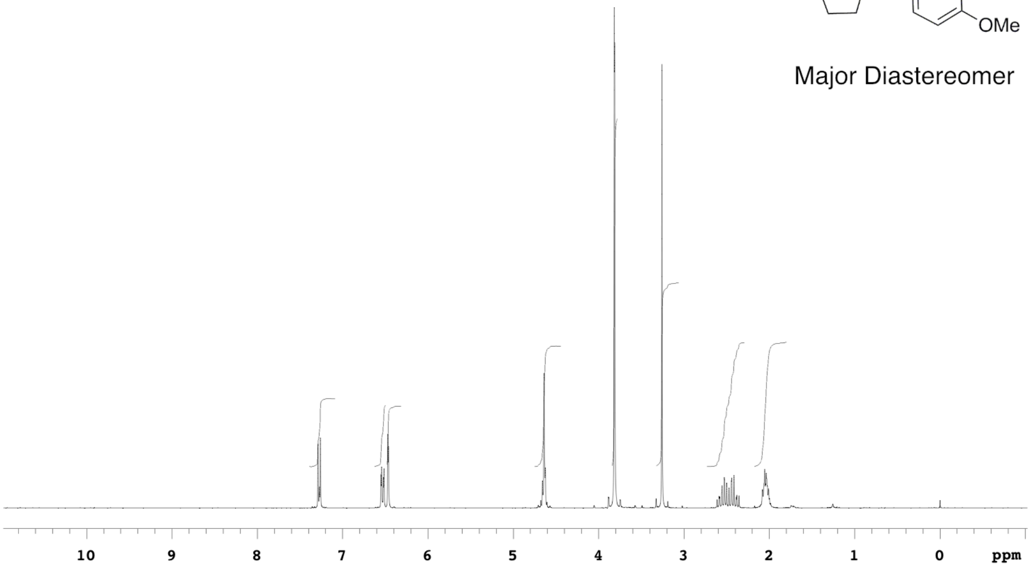

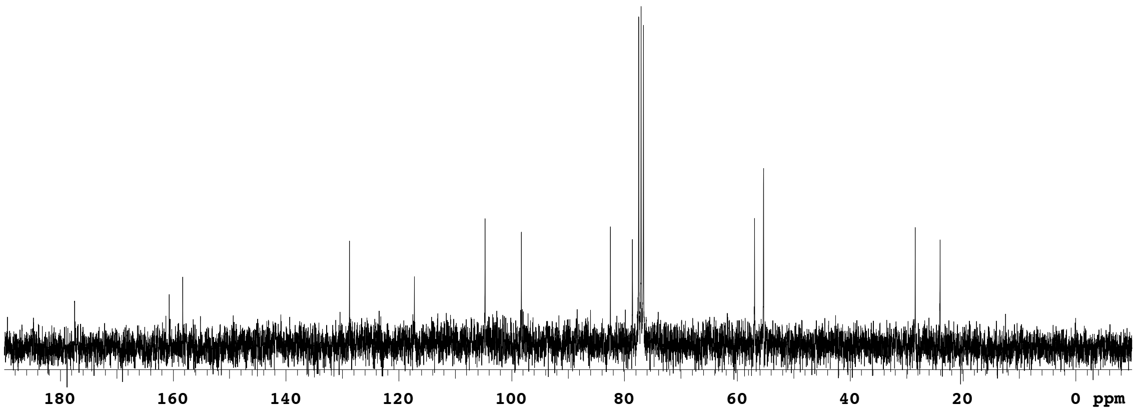

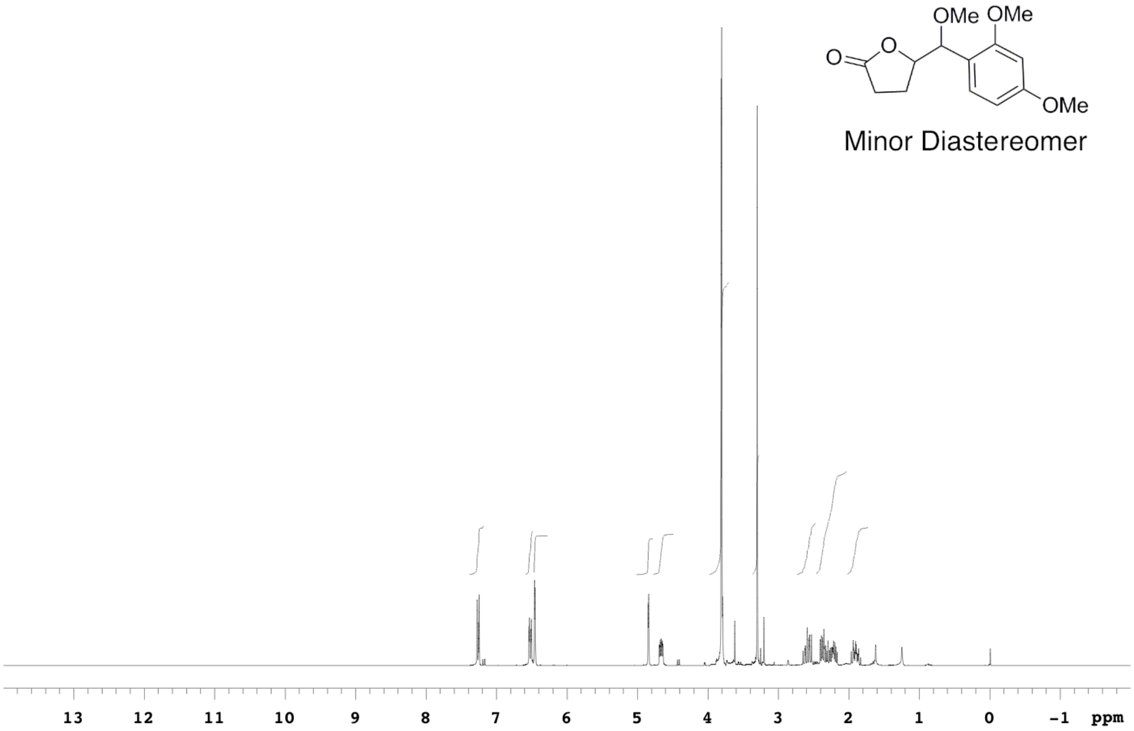

Minor Diastereomer
13 12 11 10 9 8 7 6 5 4 3 2 1 0 -1 ppm

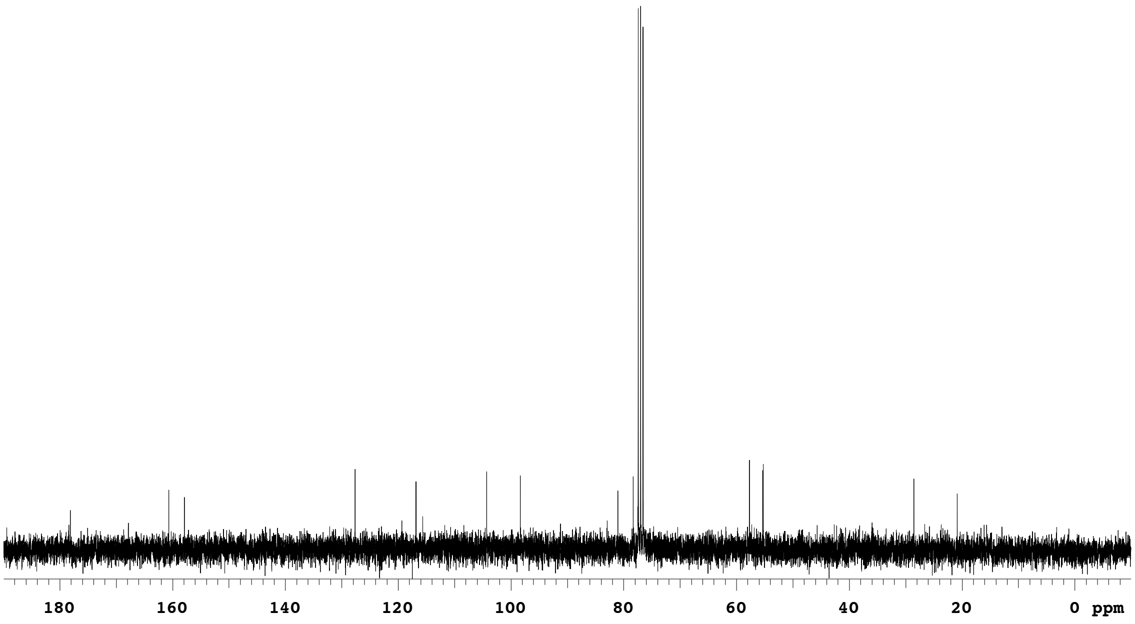

180 160 140 120 100 80 60 40 20 0 ppm

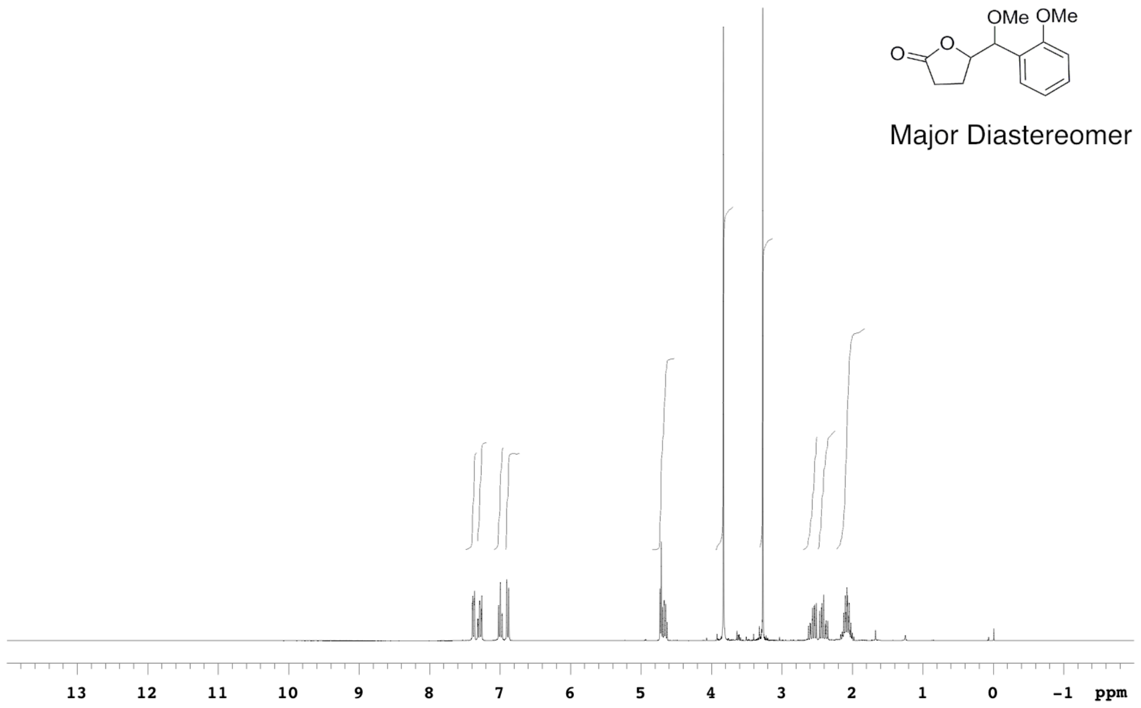
OMe OMe
O
O
Major Diastereomer
13 12 11 10 9 8 7 6 5 4 3 2 1 0 -1 ppm

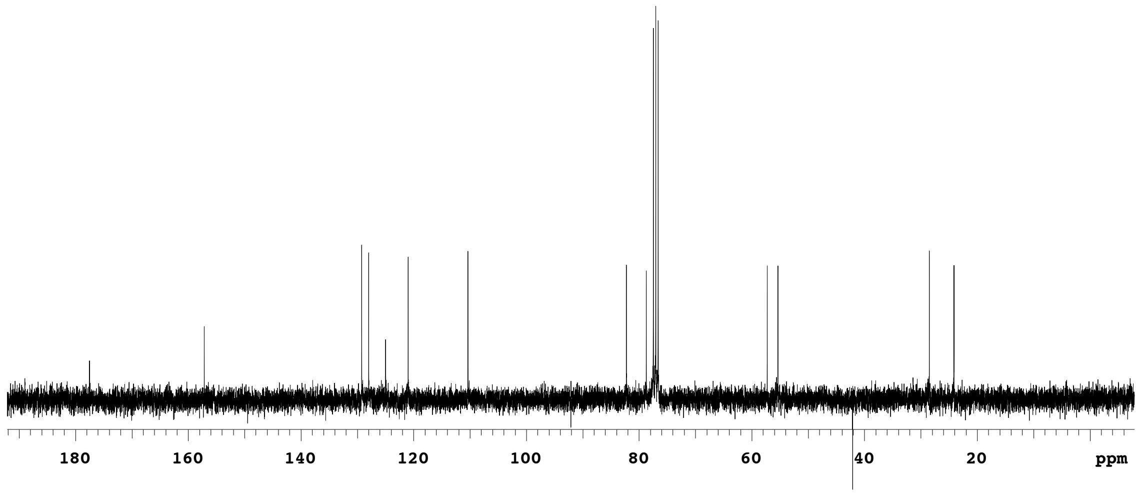
180 160 140 120 100 80 60 40 20 ppm

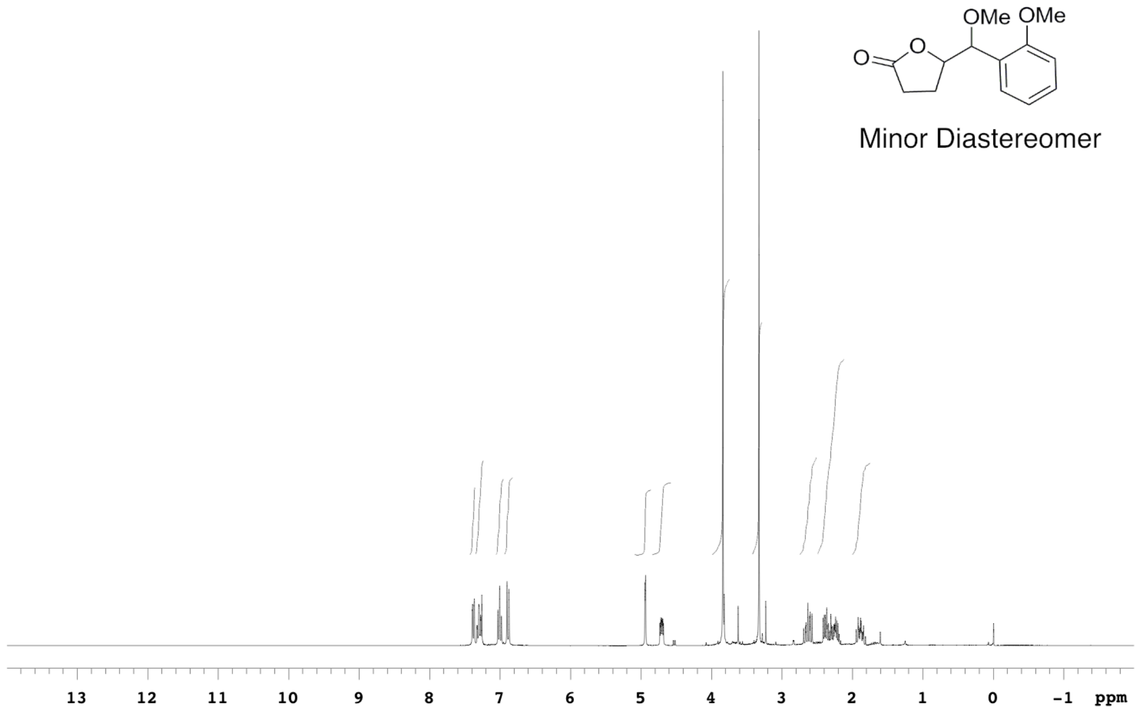
OMe
OMe
O
O
Minor Diastereomer
13
12
11
10
9
8
7
6
5
4
3
2
1
0
-1
ppm

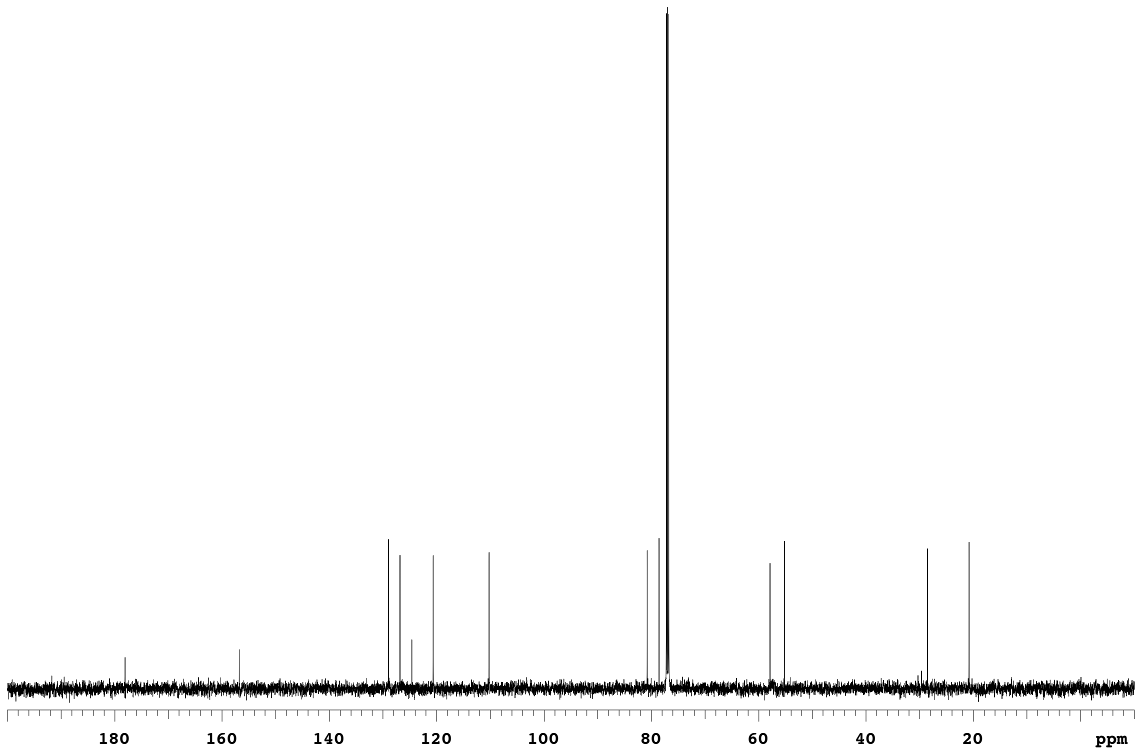
180
160
140
120
100
80
60
40
20
ppm

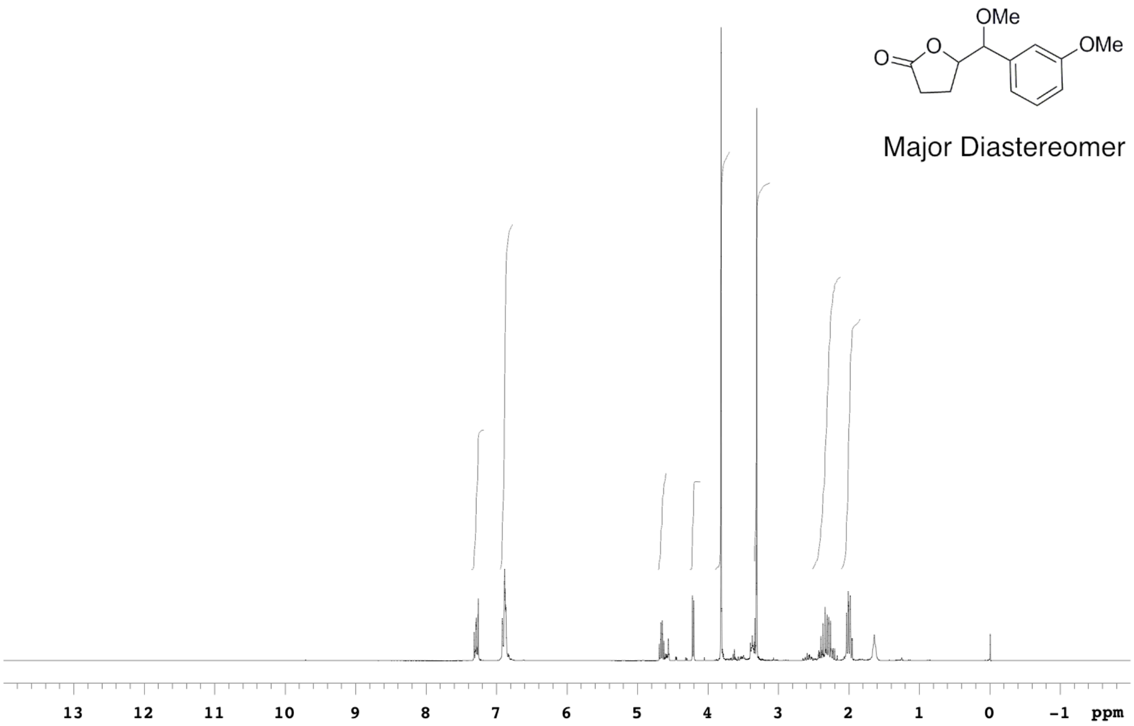
OMe
O
O
OMe
Major Diastereomer
13 12 11 10 9 8 7 6 5 4 3 2 1 0 -1 ppm

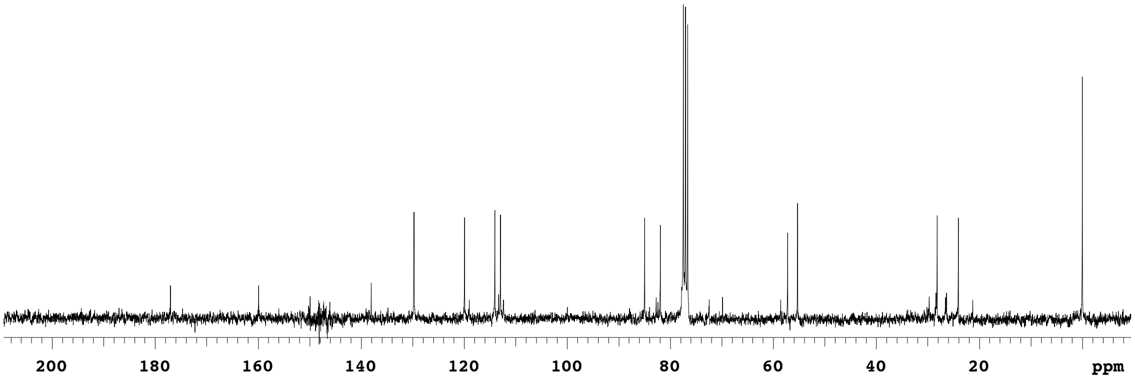
200 180 160 140 120 100 80 60 40 20 ppm

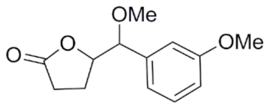

Minor Diastereomer

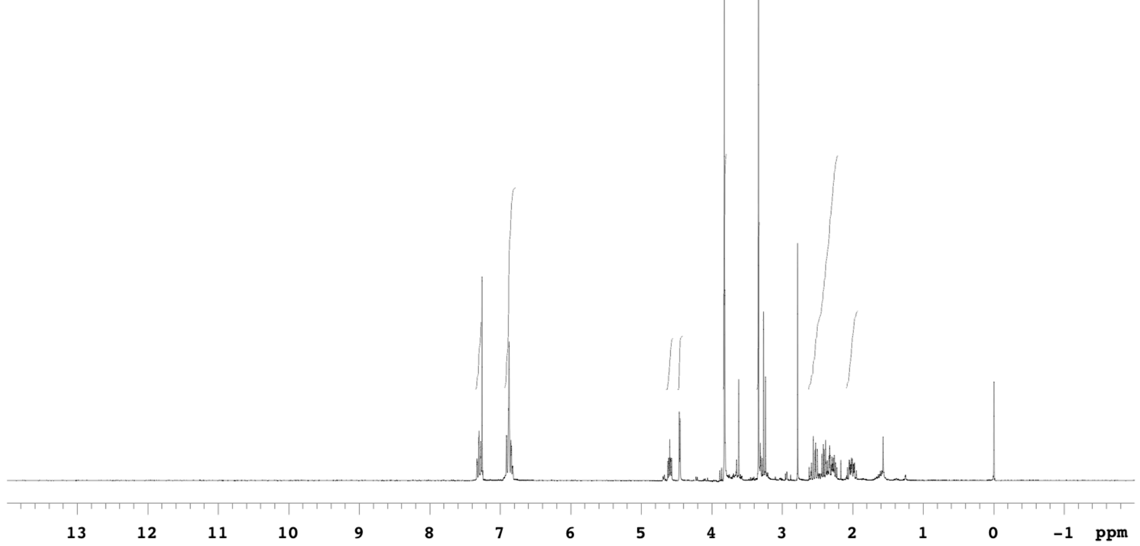

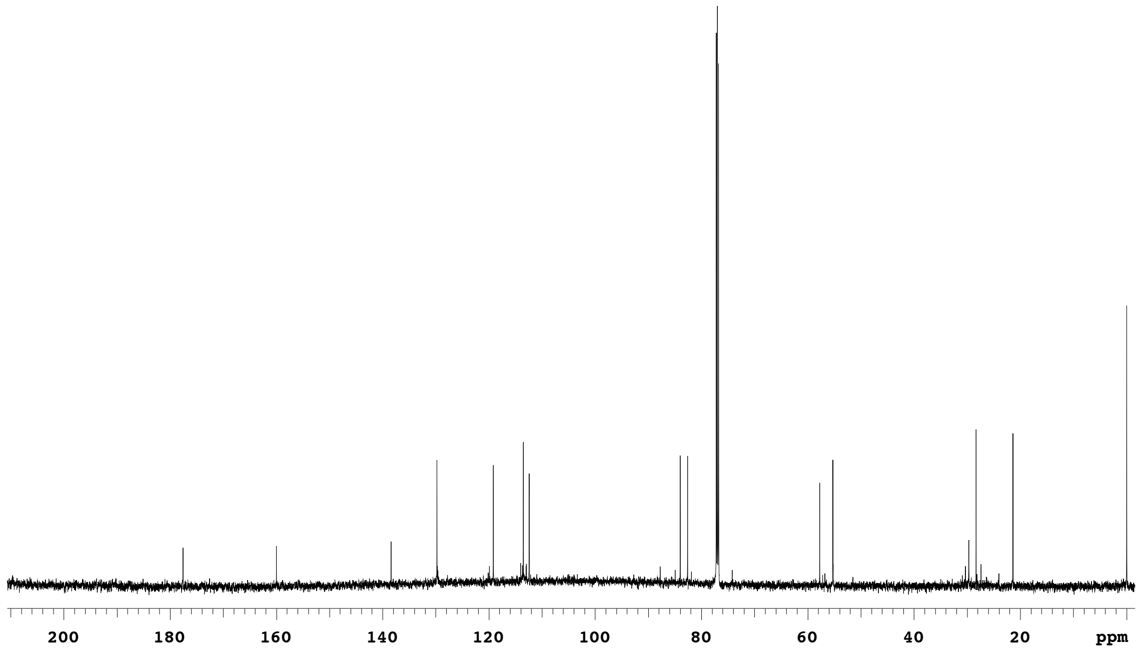

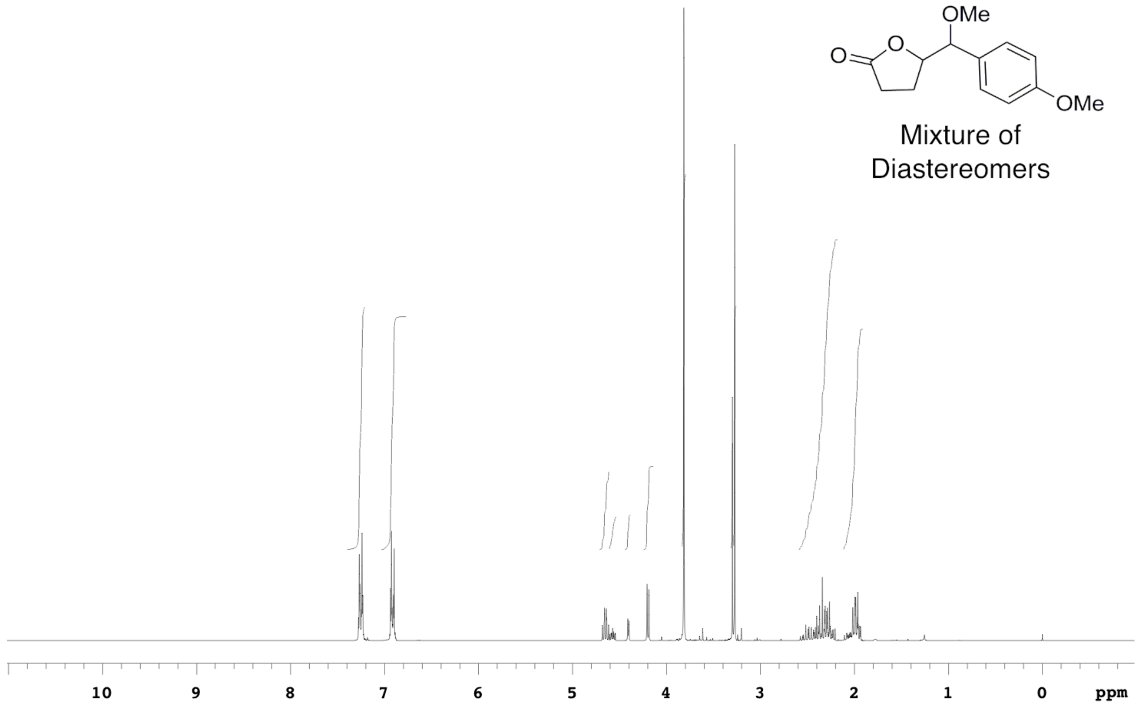
OMe
O
O
OMe
Mixture of
Diastereomers
10
9
8
7
6
5
4
3
2
1
0
ppm

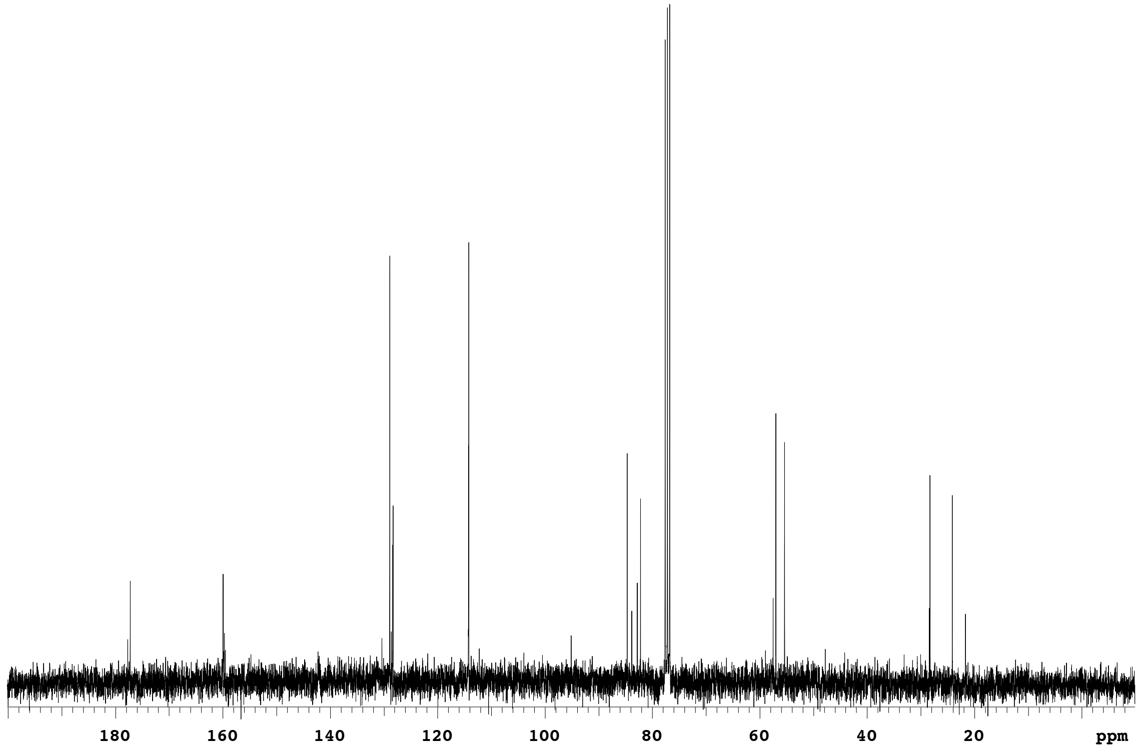
180
160
140
120
100
80
60
40
20
ppm

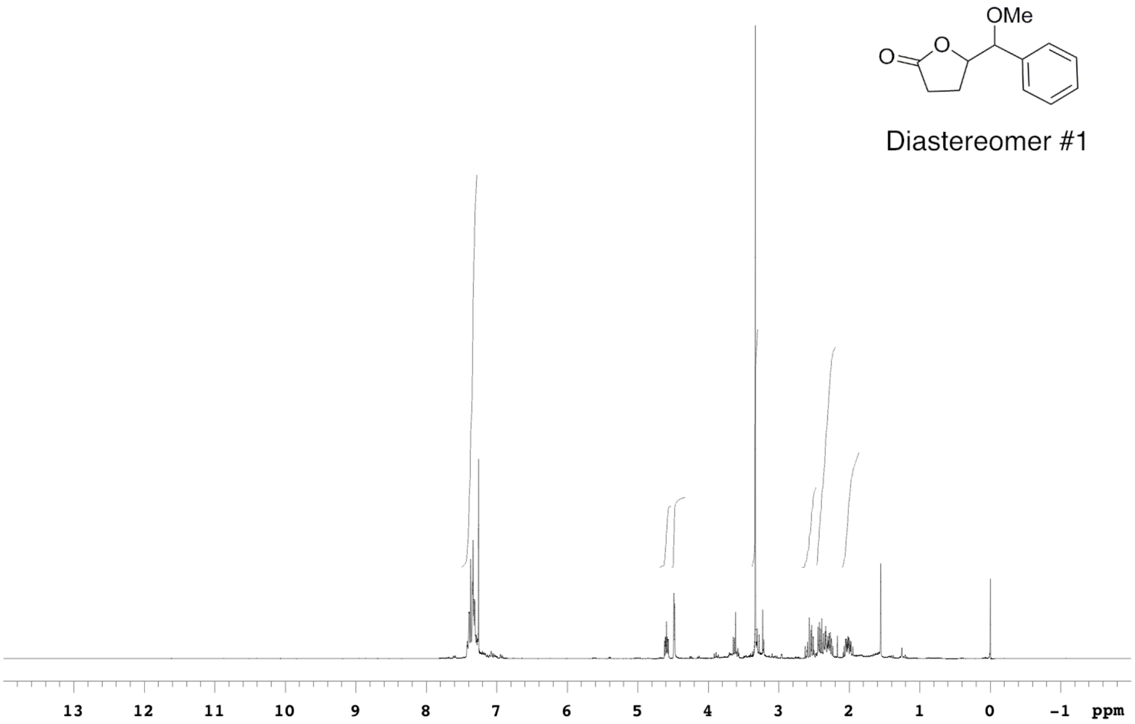
OMe
O
O
Diastereomer #1
13 12 11 10 9 8 7 6 5 4 3 2 1 0 -1 ppm

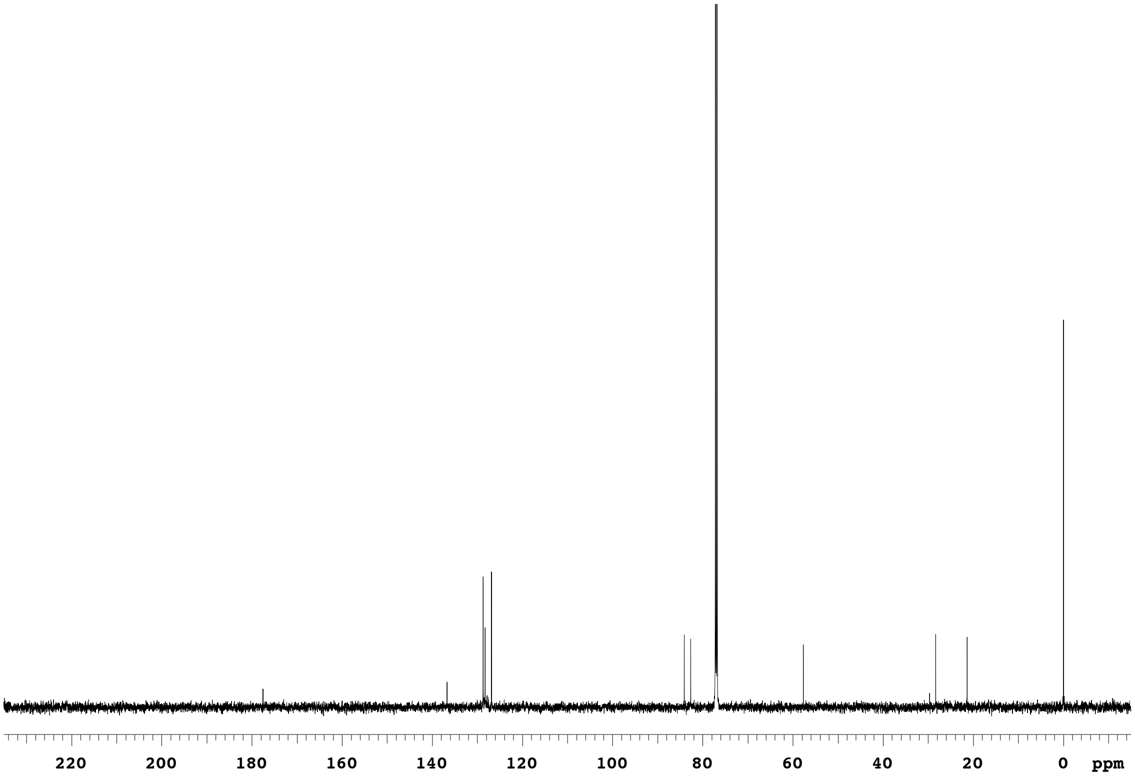
220 200 180 160 140 120 100 80 60 40 20 0 ppm

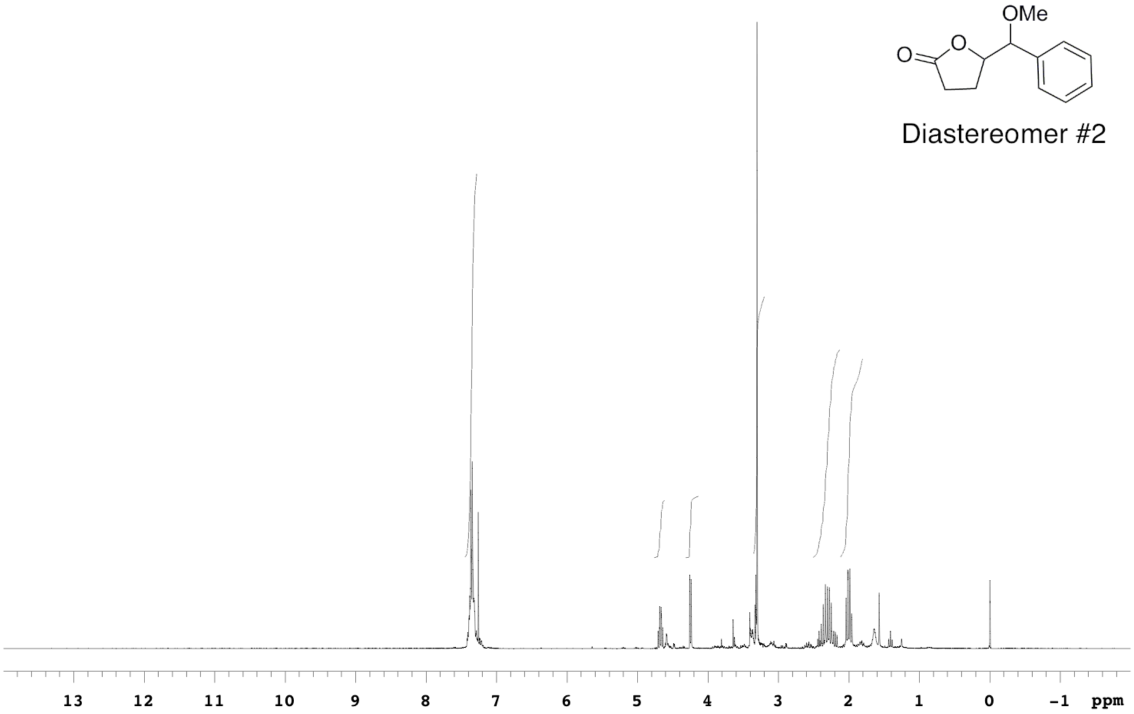
OMe
O
O
Diastereomer #2
13 12 11 10 9 8 7 6 5 4 3 2 1 0 -1 ppm

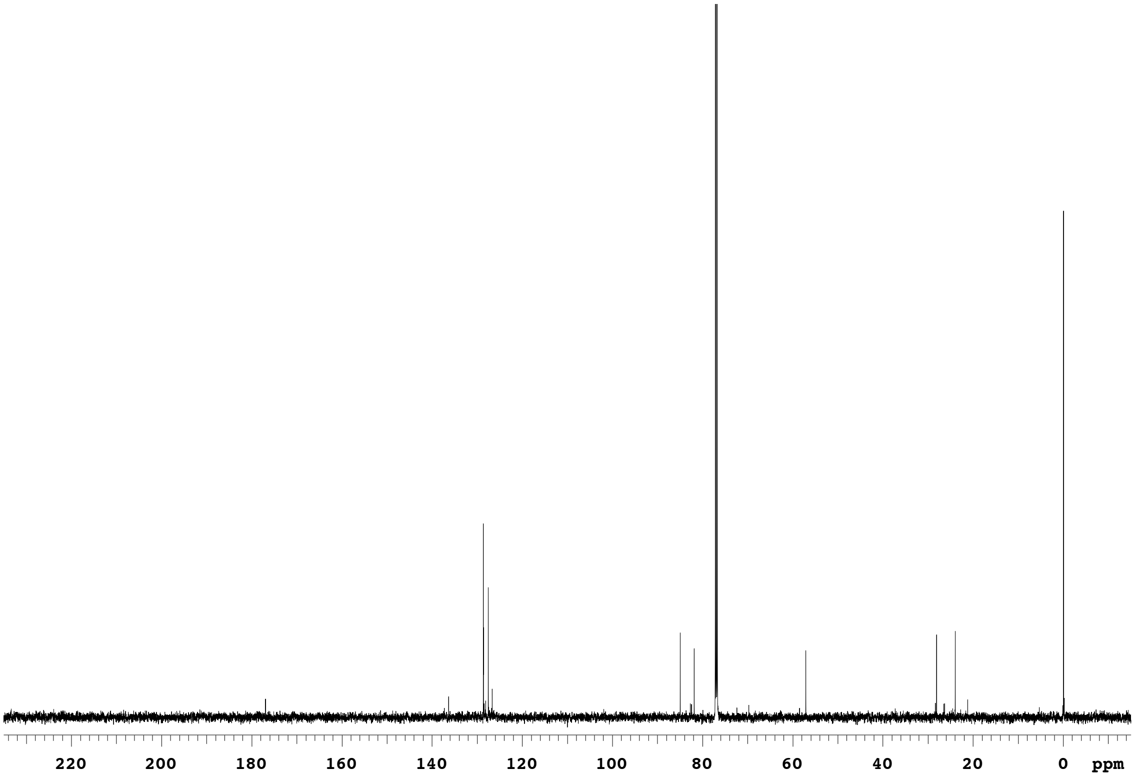
220 200 180 160 140 120 100 80 60 40 20 0 ppm

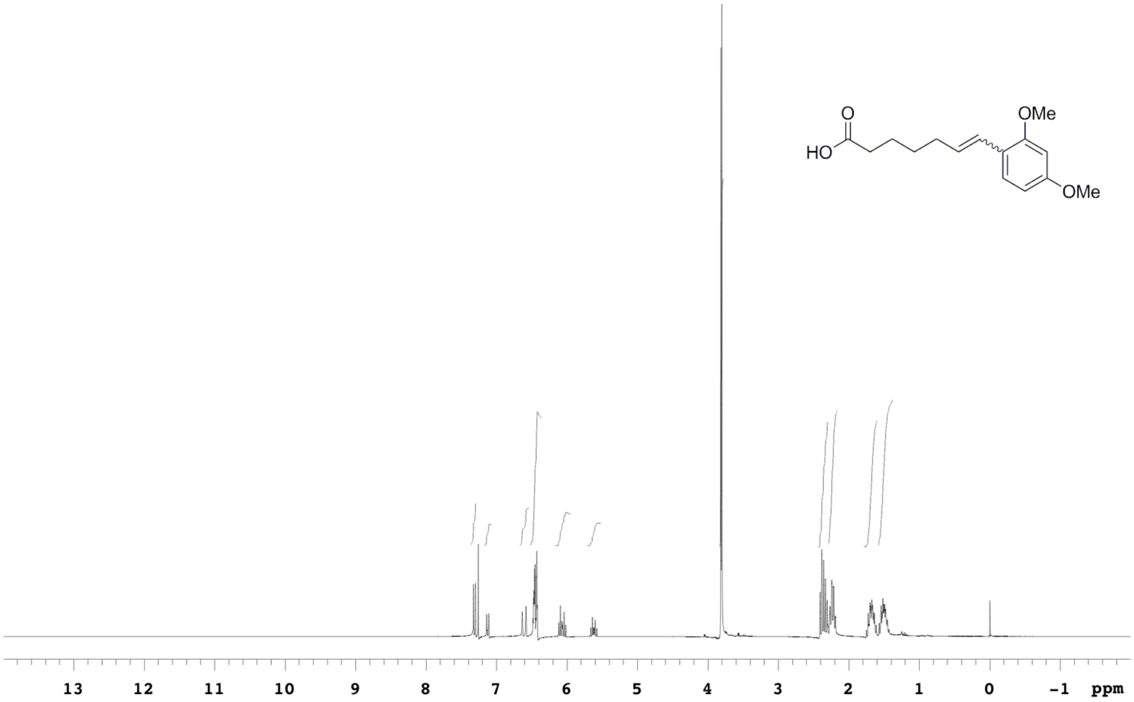
O
HO
OMe
OMe
13
12
11
10
9
8
7
6
5
4
3
2
1
0
-1
ppm

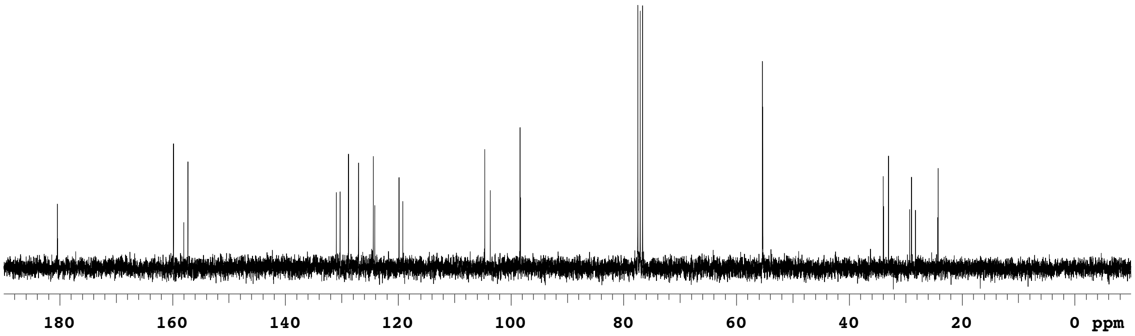
180
160
140
120
100
80
60
40
20
0
ppm

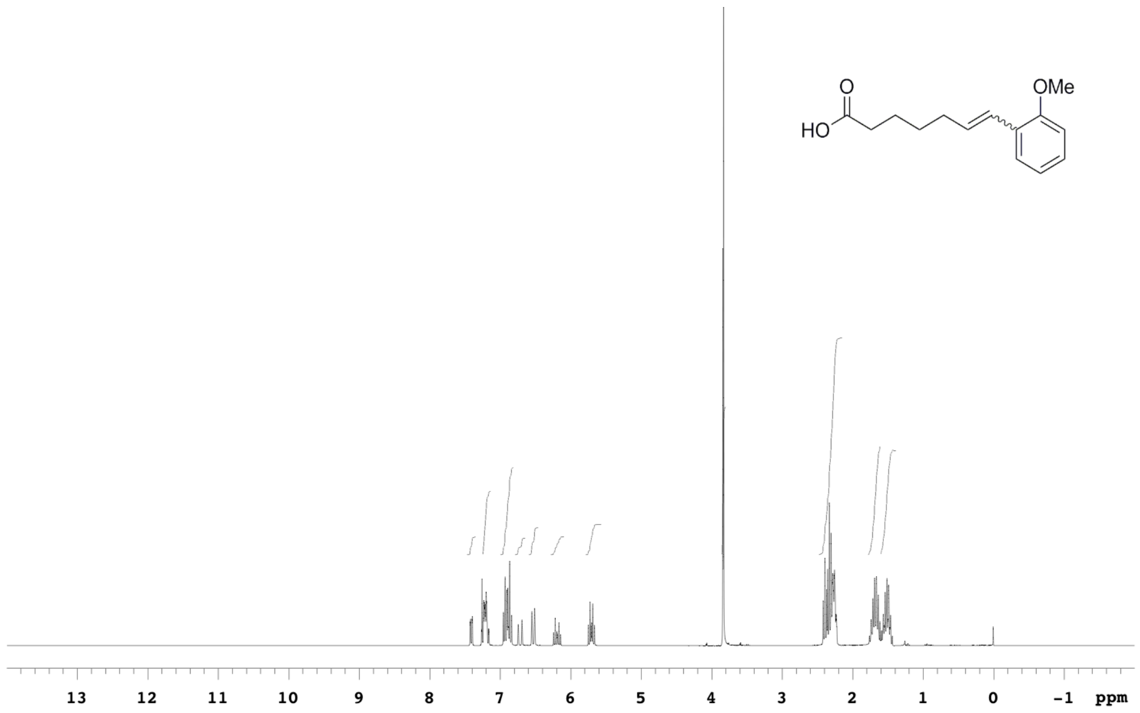
OMe
O
HO
13 12 11 10 9 8 7 6 5 4 3 2 1 0 -1 ppm

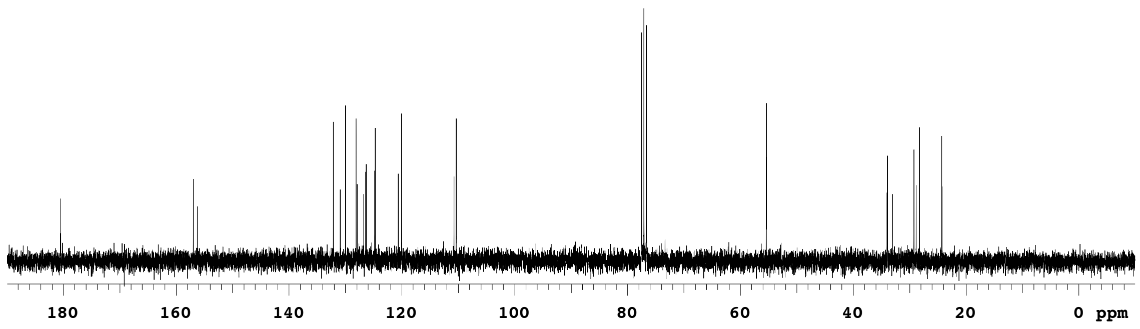
180 160 140 120 100 80 60 40 20 0 ppm

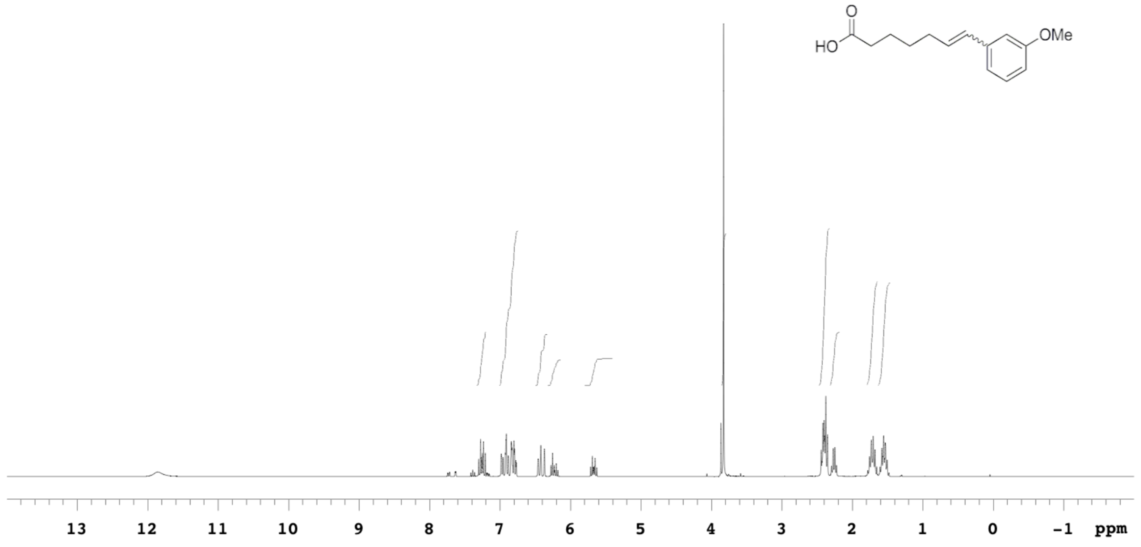
HO
O
OMe
13
12
11
10
9
8
7
6
5
4
3
2
1
0
-1
ppm

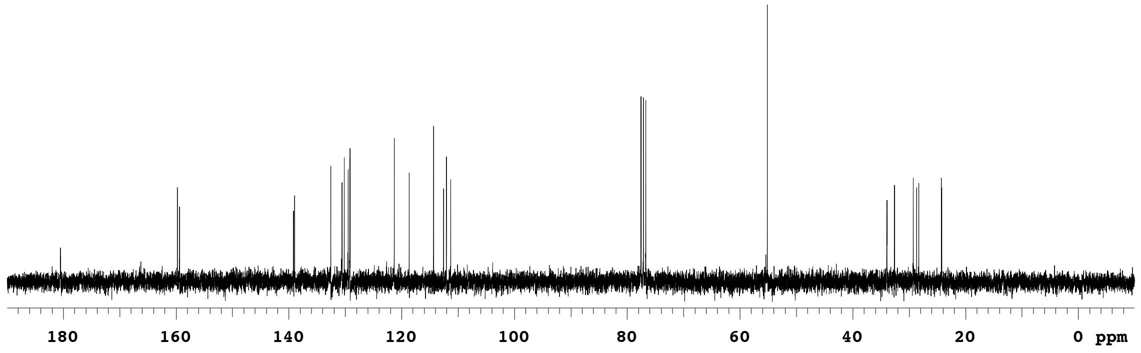
180
160
140
120
100
80
60
40
20
0
ppm
